# Combinations of the azaquinazoline anti-*Wolbachia* agent, AWZ1066S, with benzimidazole anthelmintics synergise to mediate sub-seven-day sterilising and curative efficacies in experimental models of filariasis

**DOI:** 10.3389/fmicb.2024.1346068

**Published:** 2024-02-01

**Authors:** Shrilakshmi Hegde, Amy E. Marriott, Nicolas Pionnier, Andrew Steven, Christina Bulman, Emma Gunderson, Ian Vogel, Marianne Koschel, Alexandra Ehrens, Sara Lustigman, Denis Voronin, Nancy Tricoche, Achim Hoerauf, Marc P. Hübner, Judy Sakanari, Ghaith Aljayyoussi, Fabian Gusovsky, Jessica Dagley, David W. Hong, Paul O'Neill, Steven A. Ward, Mark J. Taylor, Joseph D. Turner

**Affiliations:** ^1^Department of Tropical Disease Biology, Centre for Drugs and Diagnostics, Centre for Neglected Tropical Diseases, Liverpool School of Tropical Medicine, Liverpool, United Kingdom; ^2^Department of Pharmaceutical Chemistry, University of California, San Francisco, San Francisco, CA, Unites States; ^3^Department of Immunology and Parasitology, Institute for Medical Microbiology, University Hospital Bonn, Bonn, Germany; ^4^Laboratory of Molecular Parasitology, Lindsley F. Kimball Research Institute, New York Blood Center, New York, NY, Unites States; ^5^German Center for Infection Research (DZIF), Partner Site Bonn-Cologne, Bonn, Germany; ^6^Eisai Co., Ltd., Tokyo, Japan; ^7^Department of Chemistry, University of Liverpool, Liverpool, United Kingdom

**Keywords:** lymphatic filariasis, *Wolbachia*, onchocerciasis, AWZ1066S, benzimidazole, macrofilaricidal drugs, anti-*Wolbachia* drugs

## Abstract

Lymphatic filariasis and onchocerciasis are two major neglected tropical diseases that are responsible for causing severe disability in 50 million people worldwide, whilst veterinary filariasis (heartworm) is a potentially lethal parasitic infection of companion animals. There is an urgent need for safe, short-course curative (macrofilaricidal) drugs to eliminate these debilitating parasite infections. We investigated combination treatments of the novel anti-*Wolbachia* azaquinazoline small molecule, AWZ1066S, with benzimidazole drugs (albendazole or oxfendazole) in up to four different rodent filariasis infection models: *Brugia malayi—*CB.17 SCID mice*, B. malayi—*Mongolian gerbils, *B. pahangi—*Mongolian gerbils, and *Litomosoides sigmodontis—*Mongolian gerbils. Combination treatments synergised to elicit threshold (>90%) *Wolbachia* depletion from female worms in 5 days of treatment, using 2-fold lower dose-exposures of AWZ1066S than monotherapy. Short-course lowered dose AWZ1066S-albendazole combination treatments also delivered partial adulticidal activities and/or long-lasting inhibition of embryogenesis, resulting in complete transmission blockade in *B. pahangi* and *L. sigmodontis* gerbil models. We determined that short-course AWZ1066S-albendazole co-treatment significantly augmented the depletion of *Wolbachia* populations within both germline and hypodermal tissues of *B. malayi* female worms and in hypodermal tissues in male worms, indicating that anti-*Wolbachia* synergy is not limited to targeting female embryonic tissues. Our data provides pre-clinical proof-of-concept that sub-seven-day combinations of rapid-acting novel anti-*Wolbachia* agents with benzimidazole anthelmintics are a promising curative and transmission-blocking drug treatment strategy for filarial diseases of medical and veterinary importance.

## Introduction

Filariasis is a serious risk to health and economic prosperity in low- and middle-income countries. Lymphatic filariasis (LF) is a mosquito-transmitted disease caused by filarial nematode parasites *Wuchereria bancrofti, Brugia malayi*, or *Brugia timori* and results in debilitating pathologies, including severe lymphoedema in 36 million individuals, with further 800 million individuals estimated at risk of getting infected (Local Burden of Disease Neglected Tropical Diseases Control, 2020; WHO, 2020). Brugian filariasis reservoir infections in cats and dogs further jeopardise its elimination efforts in Asia (Nochot et al., 2019; Mallawarachchi et al., 2021), whilst the related veterinary filaria, *Dirofilaria immitis*, is the cause of heartworm disease in these companion animals and, along with *D. repens*, is a zoonotic risk to human health (Simon et al., 2017). Curative treatments of dogs are based on the arsenical drug, melarsomine, which requires a complex long-term treatment strategy in dogs with intensive veterinary management due to the risk of serious inflammatory side effects. There is no established cure for heartworm in cats (Turner et al., 2020). Onchocerciasis, caused by *Onchocerca volvulus*, is transmitted by blackflies and is the cause of skin and ocular disease (river blindness) in 14 million people.

Current efforts to eliminate human filariasis as a public health problem have focused on mass drug administration (MDA) with the established anthelmintics Ivermectin (IVM), Albendazole (ABZ), and Diethylcarbamazine (DEC) (Crump, 2017; Gyapong et al., 2018). These drugs act directly against the infective microfilarial stage or temporarily disrupt embryogenesis in the case of ABZ to remove microfilariae (mf) from circulation or skin, with the aim of breaking the transmission cycle. Because adult filarial nematode infections are chronic, and MDA drugs have negligible or low levels of curative activity against adult parasites, this strategy requires annual administrations, with high population coverage, for up to 15 years to achieve elimination. Additionally, treatment with IVM is unsuitable for patients co-infected with eye worm (*Loa loa*) and harbouring high *L. loa* mf loads due to severe life-threatening post-treatment reactions caused by the rapid microfilaricidal effect of IVM on *L. loa* mf (Gardon et al., 1997). Although MDA has successfully achieved LF elimination in some countries, the strategy has been less effective in achieving expected outcomes in India and sub-Saharan African countries (except for Togo and Malawi) (Sodahlon et al., 2013; WHO, 2019; Modi et al., 2021). Whilst onchocerciasis has been certified as eliminated from Colombia, Ecuador, Guatemala, and Mexico following biannual IVM treatment, no country in sub-Saharan Africa has yet achieved elimination despite intensive IVM MDA deployment as a front-line control and elimination strategy since 1995 (Lakwo et al., 2020; WHO, 2022). Therefore, there is a demand for new short-course drug treatments that are macrofilaricidal and can be safely employed in *L. loa* endemic regions in Africa to accelerate the elimination of human filariasis (WHO, 2020). Short-course macrofilaricides avoiding toxic or inflammatory side effects would be equally valuable in the management of veterinary/zoonotic filariasis in cats and dogs.

The causative agents of LF, onchocerciasis, and heartworm contain the endosymbiont *Wolbachia* (Slatko et al., 2010). This mutualism is essential for normal biological function, including larval development, embryogenesis, and survival, in filarial worms (Turner et al., 2020). *Wolbachia* provides an essential source of nucleotides as well as haem, riboflavin (vitamin B2), and FAD (flavin adenine dinucleotide) (Foster et al., 2005; Wu et al., 2009; Li and Carlow, 2012). *Wolbachia* occupies two distinct intra-cellular niches within filarial tissues, the hypodermal chord syncytium and the female germline, where they spread via host cell division. The germline is seeded with *Wolbachia* via infection from the hypodermal population during early fourth-stage larval development. *Wolbachia* subsequently localises in the embryonic stem cell niche by mitotic segregation (Landmann et al., 2012). Effective antibiotic depletion of *Wolbachia* induces rapid apoptosis of eggs and developing embryos, spreading to trigger apoptosis in somatic tissues of uterine mf and culminating in cessation of mf production (Landmann et al., 2011). Sterilisation of female filariae post-*Wolbachia* removal appears irreversible because in doxycycline LF and onchocerciasis clinical trials, patients remain free of mf, and excerpted *Onchocerca* female worms are devoid of uterine embryonic contents for more than 2 years post-treatment (Hoerauf et al., 2008). Removal of *Wolbachia* from hypodermal chords does not trigger widespread apoptosis at this tissue site but manifests in a more gradual decline in both female and male adult longevity, reducing life-span from 10–15 years to 18–24 months, as determined in doxycycline clinical trials (Taylor et al., 2005; Hoerauf et al., 2008).

A key advantage of a drug targeting *Wolbachia* in filariasis is that the gradual depletion of mf from blood or skin post-embryogenesis blockade avoids the inflammatory adverse reactions experienced following rapid-acting microfilaricidal treatments, particularly in *L. loa* co-infection (Haarbrink et al., 2000; Ehrens et al., 2022). An administration of doxycycline (DOX) for treating onchocerciasis in areas of *L. loa* co-endemicity has been shown to be well-tolerated (Turner et al., 2010). Doxycycline is also effective in curing circulating mf stages in brugian zoonotic filariasis and is a proven curative treatment in heartworm disease, which avoids melarsomine-like acute adverse events due to the slow-kill mode of action (Kramer et al., 2018; Nochot et al., 2019).

Because protracted doxycycline regimens of 4–5 weeks are required to mediate both sterilising and curative outcomes in LF and onchocerciasis, and because of contraindications in children and during pregnancy, new rapid-acting drugs targeting *Wolbachia* have been developed (Johnston et al., 2021). These new drugs address a target product profile of a new curative drug for onchocerciasis, which requires ideally ≤7 days of oral dosing and is ready to be advanced into clinical testing (Specht and Kaminsky, 2022). To identify novel anti-*Wolbachia* candidates, we screened 10,000 compounds using a *Wolbachia*-infected *Aedes albopictus* cell line (Clare et al., 2015; Johnston et al., 2017). From this screening, we identified and developed the first-in-class azaquinazoline, AWZ1066S (Hong et al., 2019), as a candidate anti-*Wolbachia* macrofilaricide.

We have previously demonstrated that the standard anthelmintic benzimidazole drug, albendazole, can synergise with registered antibiotics to deplete *Wolbachia* in rodent models of *B. malayi*. The drug could reduce the number of required daily treatment exposures to overcome a minimum, clinically relevant threshold of 90% *Wolbachia* depletion in female worms, mediate a block of mf production, and accelerate macrofilaricidal activity (Turner et al., 2017). In this study, we explore the extent to which combining the novel anti-*Wolbachia* azaquinazoline, AWZ1066S, with albendazole or the veterinary benzimidazole, oxfendazole (OXF), being repurposed for human use (Gonzalez et al., 2019), may reduce the treatment regimen and exposure time-frame necessary to deliver threshold efficacies in rodent models of filariasis. We further report the tissue-level tropisms during anti-*Wolbachia* depletion synergy when combining AWZ1066S with benzimidazoles.

## Materials and methods

### Drugs preparation

DOX, OXF, and ABZ were obtained from Sigma Aldrich (Dorset, UK) as dry powders. Prior to treatment, they were resuspended in the appropriate vehicle (DOX in distilled water, ABZ and OXF in 0.5% carboxymethyl cellulose, 0.5% benzyl alcohol, 0.4% Tween 80, and 0.9% NaCl). AWZ1066S, synthesised by Eisai Co., Ltd. (Tokyo, Japan), was provided as a dried powder and stored at 4°C. It was resuspended prior to treatment in a vehicle comprised of 55% PEG300, 25% Propylene glycol, and 20% water.

### *B. malayi in vivo* infection studies

**Figure 1:**
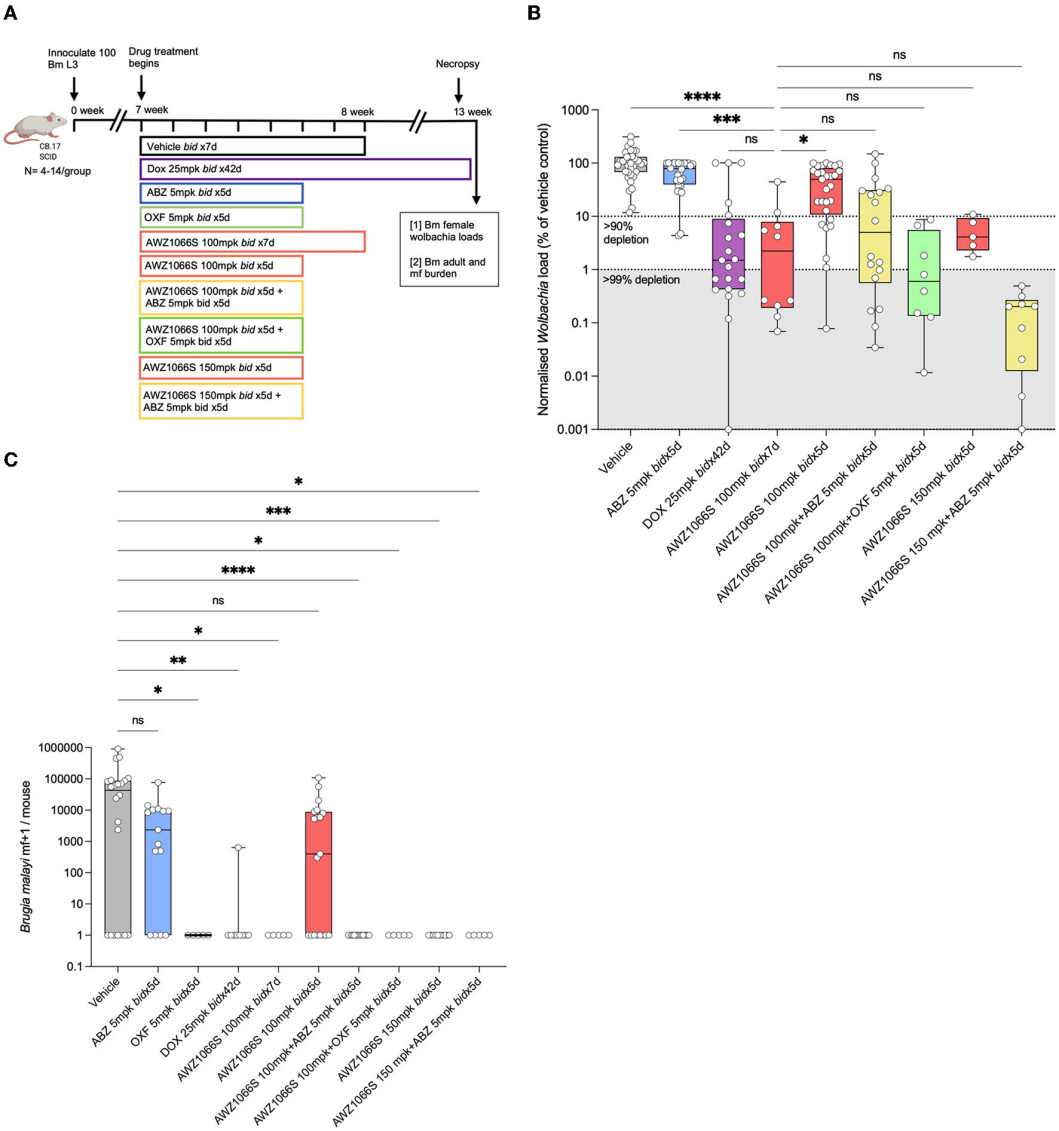
Five-day co-administration of AWZ1066S with albendazole or oxfendazole enhances filarial *Wolbachia* depletion and blocks mf production in female *B. malayi*. **(A)** Schematic of experimental design. **(B)** *Wolbachia* titres in female *B. malayi* measured by qPCR of the single copy *Wolbachia* gene, wsp. normalised percentage *Wolbachia* load compared to the median vehicle control level is plotted. **(C)** mf loads per mouse. Box and whiskers represent min/max median and interquartile range with individual plots overlayed. Significance is indicated ns, *p* < 0.05*, *p* < 0.01**, *p* < 0.001***, and *p* < 0.0001**** calculated by the Kruskal-Wallis test with Dunn's multiple comparisons. Data is derived from *n* = 5–6 mice/group from between one and four individual experiments, combined. ns, not significant.

CB.17 male severe-combined immunodeficiency (SCID) mice were purchased from Charles River (Harlow, UK). They were housed in individually ventilated cages with a HEPA-filtered air system at the University of Liverpool Biological Services Unit (UoL BSU). The mice experienced 12:12 h light:dark cycles and had access to food and water *ad libitum*. Mice were weighed before dosing, and their weight was monitored weekly following dosing to observe any decline in welfare. Male Mongolian gerbils (*Meriones ungiculatus*) were bred and maintained at UoL BSU. Animal procedures were approved by the Animal Welfare and Ethics Review Boards (AWERB) of Liverpool School of Tropical Medicine and the University of Liverpool and undertaken in accordance with UK home office licencing approval. The *B. malayi* life cycle (TRS sub-periodic human isolate) was maintained at LSTM and UoL BSU by serial passage between Mongolian gerbils and *Aedes aegypti* (Liverpool filarial susceptible strain). *B. malayi* infections in CB.17 SCID mice and gerbils were done as described previously (Turner et al., 2017; Bakowski et al., 2019; Hong et al., 2019). CB.17 SCID mice (aged 6–10 weeks) were inoculated intraperitoneally with 100 *B. malayi* third-stage (L3) larvae. Mice were weighed, and 100 μl oral drug doses were adjusted accordingly (±8 μl for each 1 g weight change from 25 g). Groups of 4–12 mice received assigned drug treatment by oral gavage commencing 6 weeks after inoculation (Figure 1A). Mice received either 100 or 150 mg/kg of AWZ1066S for 5 days two times daily (*bid*—bis in die) alone or in combination with 5 mg/kg ABZ or OXF *bid* for 5 days. Separate control groups of ABZ or OXF alone were also included, where animals received 5 mg/kg bi-daily dosing for 5 days. Sham animals only received vehicle treatment. DOX treatment with 25 mg/kg *bid* for 42 days was also included, which has been previously reported to deplete *Wolbachia* levels > 90% (Turner et al., 2017). To match the same frequency and volume of dosing between the groups, animals without additional ABZ treatment received a corresponding volume of the ABZ-vehicle and animals without AWZ1066S dosing received a matching volume of AWZ1066S-vehicle. Mice were necropsied at 6–7 weeks after commencement of treatment by rising concentration CO_2_ asphyxiation. Death was confirmed by exsanguination via cardiac puncture (Home office schedule 1 procedure). *B. malayi* adults and mf were recovered by peritoneal washing. Individual motile parasites were enumerated by microscopy and kept frozen at −20°C until further molecular analysis.

### *B. pahangi in vivo* infection studies

Male Mongolian gerbils (*Meriones unguiculatus*) aged 5–7 weeks were purchased from Charles River International (Massachusetts, USA). The study was carried out at the University of California, San Francisco and all animal procedures were approved by the University of California, San Francisco Institutional Animal Care and Use Committee (IACUC) (approvals: AN109629-03 and AN173847-02) and adhered to the guidelines set forth in the NIH guide for the care and use of laboratory animals and the USDA animal care policies. Gerbils were injected intraperitoneally (IP) with 200 *B. pahangi* L3 as described elsewhere (Gunderson et al., 2020; Hubner et al., 2020a). Twenty-four weeks post-infection, the gerbils were treated with either 50 or 100 mg/kg AWZ1066S alone or in combination with ABZ twice daily by oral gavage. ABZ was used at a 10 mg/kg bi-daily dose equilibrated to match systemic exposure of the active metabolite, ABZ-sulfoxide, as standard human 400 mg dosing in gerbils. Sham animals only received vehicle treatment. Another control group for ABZ alone was also included, where animals were treated with 10 mg/kg ABZ two times daily. To ensure that all animals are treated with the same frequency and volume, the groups without additional ABZ treatment received a corresponding volume of the ABZ-vehicle and groups without AWZ1066S dosing received a matching volume of AWZ1066S-vehicle. Animals were euthanised 19 weeks following the primary treatment (43 weeks post-infection), and the parasites were collected from the peritoneal cavity. The recovered adult worms were sexed under a dissecting microscope, counted, and kept frozen until further downstream molecular analysis. Mf in the peritoneal cavity were quantified by mixing peritoneal wash 9:1 (v/v) with 0.04% methylene blue:water and counted under an inverted microscope.

To quantify the number of mf shed after overnight culture *in vitro*, individual female worms from control and different treatment groups were cultured in RPMI medium containing 10% FBS, 2x penicillin and streptomycin at 37°C and 5% CO_2_ overnight. The next day, the number of mf released from each female was quantified by counting under the microscope.

### *L. sigmondontis in vivo* infection studies

Female Mongolian gerbils (*M. unguiculatus*) were purchased from Janvier Labs (Saint-Berthevin, France) and housed in individually ventilated cages with access to food and water *ad libitum* at the Institute of Medical Microbiology, Immunology and Parasitology, University Hospital Bonn, Germany. Housing conditions and all study procedures were performed according to the European Union animal welfare guidelines and the State Office for Nature, Environment and Consumer Protection, Cologne, Germany (AZ 84-02.04.2015.A507). *L. sigmondontis* infections in gerbils were carried out as described before (Hubner et al., 2019a,b, 2020b). Briefly, 9-week-old female jirds were naturally infected by exposure to mites *(Ornithonyssus bacoti)* containing *L. sigmodontis* L3 larvae. The treatment started 14 weeks post-infection, and animals received different doses of either AWZ1066S (25, 50, or 100 mg/kg) alone or in combination with 10 mg/kg Albendazole two times a day by oral gavage. Control groups were treated with ABZ alone (10 mg/kg, 5 d, *bid*) or vehicle alone (Sham). To match the dosing volume and frequency, animals that received a single drug dose also received vehicle treatment corresponding to the second drug. Peripheral mf levels were quantified before treatment (12 wpi) and every other week after treatment (2, 4, 6, 8, 10, 12, 14, and 17 wpt) by microscopy. Necropsy was performed 17 weeks post-treatment start. Mf was quantified by counting under the microscope from pleural lavage after diluting 10 μl of peripheral blood in 190 μl Hinkelmann solution (0.5% eosin Y, 0.5% phenol, 0.185% formaldehyde in water). If there were < 50 mf in 10 μl, the sample was centrifuged at 400 g for 5 min, the supernatant was discarded, and the pellet was resuspended and completely transferred to a microscope slide for counting. Adult worms were extracted from the thoracic cavity and peritoneum. Isolated worms were sorted according to their sex, separated and individually frozen for subsequent *Wolbachia* analysis.

Embryogram from adult worms was done as described elsewhere (Ziewer et al., 2012; Risch et al., 2023). Briefly, worms were fixed using 4% formaldehyde for 24 h and then stored in 60% ethanol until analysis. Single female worms were homogenised in 80 μl PBS and 20 μl Hinkelmann solution [0.5% (wt/vol) eosin Y, 0.5% (wt/vol) phenol (both from Merck) and 0.185% (vol/vol) formaldehyde (Sigma-Aldrich) in deionised water] was added. Ten microlitre of serially diluted worm homogenate were analysed by microscopy, and the numbers of the embryonic stages like “egg,” “morula,” “pretzel,” and “stretched mf” per female adult worm were calculated. For undiluted samples that did not contain any embryonal stages, the homogenate was centrifuged at 400 g for 5 min, and the pellet was resuspended in 10 μl and analysed by microscopy. If embryonal stages were present, five intact female worms were analysed per gerbil. The analyses were performed as previously described (Ziewer et al., 2012).

### Molecular assays

Genomic DNA was isolated from frozen worms using the DNeasy blood and tissue kit (QIAGEN, Manchester, UK) for *L. sigmodontis* via the QIAamp DND mini kit (QIAGEN, Hilden, Germany) as per the manufacturer's instructions. For *B. malayi* and *B. pahangi* infections, the single-copy *Wolbachia* gene *wsp* was measured using qPCR as previously described (Halliday et al., 2014; Sharma et al., 2016). For *L. sigmodontis* infection*, Wolbachia* numbers were quantified by qPCR using the single-copy LsFtsZ gene as previously described (Schiefer et al., 2012, 2020).

### Fluorescent *in-situ* hybridisation and microscopy analysis

*Wolbachia* detection using FISH staining was carried out as described before (Dodson et al., 2014; Walker et al., 2021) with some modifications. Frozen whole worms were fixed overnight at 4°C in ethanol and 1x PBS (1:1). Worms were incubated for 5 min at 4°C in 4% paraformaldehyde in 1X PBS and washed in 1x PBS two times. Worms were then incubated for 10 min at 37°C with a 10 μg/ml pepsin solution and washed two times in 1X PBS. Hybridisation was conducted in dark conditions at 37°C for overnight, with 100 μl of hybridisation buffer [50% formamide, 5xSSC, 200 g/L dextran sulphate, 250 mg/L poly(A), 250 mg/L salmon sperm DNA, 250 mg/L tRNA, 0.1 M dithiothreitol (DTT), and 0.5 × Denhardt's solution] containing *Wolbachia* specific 16S rRNA probes W1:/5ATTO590N/AATCCGGCCGARCCGACCC, and W2:/5ATTO590N/CTTCTGTGAGTACCGTCAT TATC. After hybridisation, worms were washed in 100 μl of washing buffer (hybridisation buffer without probes) at 37°C for 15 min. Subsequently, two washes in 1X SSC buffer with 10 mm DTT and two washes in 0.1X SSC buffer with 10 mm DTT were performed, followed by one wash in 1X PBS at room temperature. Finally, samples were mounted in Vectashield anti-fading medium with DAPI (Vector Laboratories, London, UK), stored at 4°C overnight, and then observed using a laser scanning confocal microscope (Zeiss, Cambridge, UK). No-probe controls were also included as negative controls.

For quantification of *Wolbachia* intensity, five random pictures were taken from the distal ovary and hypodermal chords from each worm. A minimum of three worms from each treatment group were imaged unless specified. *Wolbachia* intensity was measured using the ROI method in a defined unit area by ImageJ/FiJi software (https://imagej.nih.gov/ij/).

### Pharmacokinetic studies and bioanalysis

Pharmacokinetic studies were conducted to determine the drug concentrations of 10 mg/kg ABZ and 50 mg/kg AWZ1066S administered orally as monotherapies or in combination for 5 days from the *L. sigmodontis* infection model in jirds. This was done to ensure that drug exposures after combination therapies were in line with those observed in the previous rich PK studies during mono therapies (Turner et al., 2017; Hong et al., 2019). For sparse PK analysis, 8 μL blood samples were collected at 0.5, 1, 3, 6, and 24 h after first dosing vena saphena and were immediately transferred to DBS cards (Whatman 903 Protein saver card, Sigma-Aldrich, Germany). The whole blood concentration of AWZ1066S was quantified at WuXi Apptech (Couvet, Switzerland) using liquid chromatography and mass spectroscopy on a UPLC (ultrahigh-pressure liquid chromatography) as described elsewhere (Hong et al., 2019; Turner et al., 2017).

### Statistical analysis

The continuous variables of *wsp* copy numbers (in *B. malayi* and *B. pahangi*), *LsFtsZ* copy numbers, peripheral mf numbers, adult parasite counts, and embryo counts in *L. sigmodontis* did not satisfy the assumption of normal distribution by D'Agostino and Pearson omnibus tests. Statistical significance was assessed using GraphPad Prism (version 9.5.1) by Kruskal–Wallis 1-way- ANOVA tests followed by Dunn's multiple comparisons *post-hoc* test to compare three or more groups. Significant differences in *Wolbachia* signal intensity in *B. malayi* hypodermal chords and ovarian tissue following drug treatments were analysed using 2-way-ANOVA followed by Šídák's multiple comparisons *post-hoc* test.

## Results

### Five-day co-administration of AWZ1066S with albendazole or oxfendazole enhances filarial *Wolbachia* depletion and blocks mf production

AWZ1066S is a first-in-class azaquinazoline anti-*Wolbachia* small molecule that can deliver >90% *Wolbachia* depletion following 7-day oral exposures in pre-clinical animal models compared to 4–6 weeks needed to achieve the same level of depletion by DOX (Turner et al., 2017; Hong et al., 2019). In a CB.17 SCID mouse model of brugian filariasis (Halliday et al., 2014), we previously defined a dose of 100 mg/kg given two times daily (*bid*) for seven days as minimally effective in driving >90% depletions in adult female *Wolbachia* loads and knock-on disruption of embryogenesis leading to prevention of mf production (Hong et al., 2019). To test whether albendazole (ABZ) could synergise with AWZ1066S to boost *Wolbachia* depletion, we treated *B. malayi-*infected CB.17 SCID mice with AWZ1066S alone or in combination with ABZ two times daily for 5 days (Figure 1A). Bi-daily 25 mg/kg DOX treatment (matching 100 mg daily dosing in humans) (Sharma et al., 2016) for 6 weeks was used as a reference control that resulted in 99.3% median *Wolbachia* depletion in female *B. malayi* compared to vehicle (Figures 1B, C). ABZ treatment at 5 mg/kg two times daily for 5 days [bioequivalent to 400 mg standard daily dosing in humans (Turner et al., 2017)] did not significantly alter *Wolbachia* loads. AWZ1066S 100 mg/kg bi-daily dosing for 7 days resulted in an expected >90% *Wolbachia* efficacy (median 97.8% depletion). When we reduced this regimen to 5 bi-daily cycles of 100 mg/kg, this was significantly less effective at depleting *Wolbachia* in female *B. malayi* (a 51.4% reduction, Kruskal-Wallis 1-way-ANOVA *p* < 0.0001, Dunn's *post-hoc* test *p* < 0.05 vs. AWZ1066S 100 mg/kg *bid* x7). However, co-administration of 5 mg/kg ABZ with 100 mg/kg of AWZ1066S for 5 days rescued this regimen in terms of mediating a >90% significant reduction in *Wolbachia* levels (98.8% median depletion) which was equipotent compared with a 7-day dosing (Figure 1B). We further tested whether oxfendazole (OXF), another benzimidazole drug with improved oral bioavailability, could also mediate synergy when co-administered with AWZ1066S. We observed low or absent adult *B. malayi* worm burdens and mf in oxfendazole-treated animals (Figure 1C; Supplementary Table S1), consistent with direct macrofilaricidal activity defined in other filariasis research models (Hubner et al., 2020b). In surviving worms, combined bi-daily dosing of 5 mg/kg OXF with 100 mg/kg AWZ1066S for 5 days also enhanced the level of *Wolbachia* depletion efficacy to >99%, demonstrating synergy is not specific to ABZ but rather consistent with a class-wide effect of benzimidazole co-administration (Figure 1B). When we co-administered elevated 150 mg/kg bi-daily doses of AWZ1066S for 5 days, we could achieve a potency boost of 96 vs. 51.4% median *Wolbachia* depletions. When co-dosed with ABZ, this efficacy increased to 99.8% depletion, further illustrating the synergistic potential of ABZ co-administration.

The transmission-blocking impact of combination synergy was examined by enumerating the total numbers of *B. malayi* mf within the peritoneal cavity of infected SCID mice (Figure 1C). In vehicle control animals, 7/22 infections were mf negative (32%), and the median yield of mf/mouse was 4.3 × 10^4^. ABZ had reduced but not prevented mf accumulations in the majority of mice (4/15 mice mf-, median 0.5 × 10^4^), and mf production was not significantly different from vehicle control levels. Six-week bi-daily DOX or 7-day AWZ1066S at 100 mg/kg significantly prevented mf release, whereby 8/9 and 5/5 mice were mf negative, respectively (Kruskal-Wallis 1-way-ANOVA *p* < 0.0001, Dunn's *post-hoc* tests *p* = 0.0005 and *p* = 0.0092, respectively). The reduced 5-day bi-daily dosing of AWZ1066S at 100 mg/kg failed to block mf release in the majority of mice treated (8/19 mice mf-, median yield of 0.04 × 10^4^), and mf production in this treatment group was not significantly different to those in the vehicle control group. Comparatively, when ABZ or OXF was added to this regimen as a combination, mf production was completely blocked (15/15 and 5/5 mice, Dunn's *post-hoc* tests, *p* < 0.0001 and *p* = 0.003, respectively). The elevated 150 mg/kg dose of AWZ1066S two times-daily for 5 days achieved a complete block of mf production regardless of whether ABZ was co-dosed (7/7 and 5/5 mice, Dunn's *post-hoc* tests, *p* < 0.0001 & *p* = 0.003, respectively). There was no significant difference in total adult worm burden among the control and treatment groups (Supplementary Table S1). In summary, our data demonstrates it is feasible to mediate >90% *Wolbachia* depletion and block mf production from female *B. malayi* worms following sub-seven-day dosing of AWZ1066S. Combinations with either benzimidazole, ABZ, or OXF synergise to reduce the dose exposure of AWZ1066S necessary to mediate these threshold anti-*Wolbachia* and sterilising activities.

### Five-day combinations with albendazole reduce the dose exposure of AWZ1066S necessary to deliver long-term anti-*Wolbachia* anti-filarial activities

**Figure 2:**
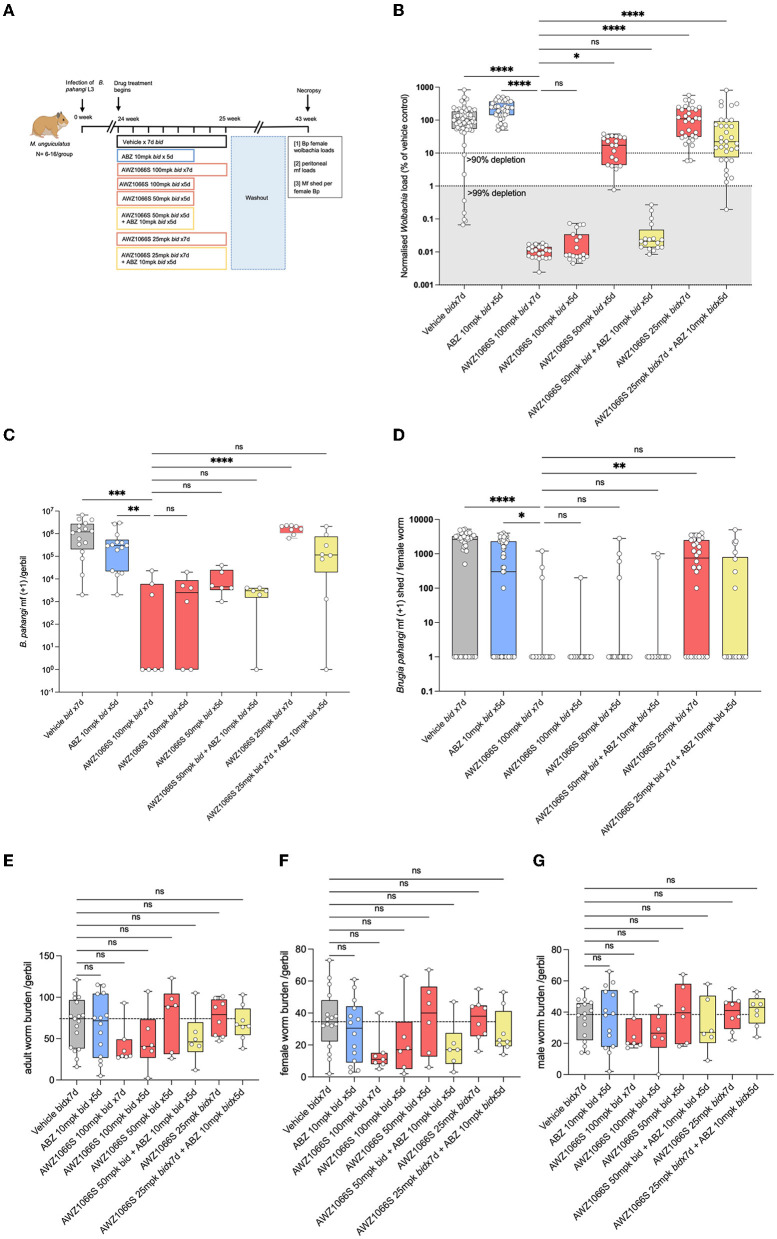
Synergistic depletion of *Wolbachia* and mf blockade in female *B. pahangi* after low-dose 5-day AWZ1066S and ABZ combination therapy. **(A)** Schematic representation of the experimental design; **(B)** *Wolbachia* numbers in individual female *B. pahangi* measured by qPCR for the single copy *Wolbachia* gene wsp; **(C)** the total number of mf recovered from gerbil peritonea; and **(D)** the number of mf shed from individual female worms cultured overnight. Enumeration of total **(E)**, female **(F)**, and male **(G)** adult worm burdens at end-point. Box and whiskers indicate min/max, median, and interquartile range with individual data overlayed. Significance is indicated: ns, *p* < 0.05*, *p* < 0.01**, *p* < 0.001***, and *p* < 0.0001**** calculated by the Kruskal-Wallis test with Dunn's multiple comparisons. Data is derived from *n* = 6–8 gerbils/group from two individual experiments combined. ns, not significant.

To confirm that the “rescue” effect of co-treating lowered 5-day doses of AWZ1066S with benzimidazoles was not unique to *B. malayi* and to verify the persistence of efficacy in terms of potential recrudescence of *Wolbachia* and resumption of mf production, we utilised long-term models of lymphatic filariasis in gerbils. The *B. pahangi* infection model was used to test the effect of AWZ1066S and ABZ combination on *Wolbachia* depletion in mature, fecund female worms. We infected *Meriones unguiculatus* Mongolian gerbils with *B. pahangi* L3, and following 24 weeks of infection, gerbils were treated bi-daily with different concentrations of AWZ1066S either alone or in combination with 10 mg/kg ABZ two times daily (Figure 2A). This dose of ABZ was determined to be bio-equivalent to SCID mouse dosing at 5 mg/kg (Supplementary Figure S1) and thus aligned with 400 mg daily exposures in humans (Shenoy et al., 2002; Turner et al., 2017; Ceballos et al., 2018). Gerbils were necropsied after a long washout period of 17 weeks (Figure 2A). The quantification of *wsp* copies in female worms by qPCR confirmed the SCID mouse model data in *B. malayi*, that a bi-daily treatment of 100 mg/kg AWZ1066S as a monotherapy for 7 days was sufficient to result in >99% reduction in *Wolbachia* titres in adult female worms, whereas a 5-day ABZ monotherapy did not have any anti-*Wolbachia* efficacy (Figure 2B). In this model, *B. pahangi* females were more sensitive to the anti-*Wolbachia* effect of 5-day bi-daily AWZ1066S at 100 mg/kg two times daily than in the *B. malayi* SCID model (Figure 1). This regimen resulted in >99.9% *Wolbachia* depletion irrespective of ABZ co-dosing, which was statistically non-inferior to the corresponding 7-day AWZ1066S regimen (Figure 2B). However, when we de-escalated a 5-day dosing of bi-daily AWZ1066S to 50 mg/kg, monotherapy mediated <90% *Wolbachia* depletion, which was inferior to a 7-day 100 mg/kg dosing (82.7% median depletion, Kruskal-Wallis 1-way-ANOVA *p* < 0.0001, Dunn's *post-hoc* test *p* = 0.015). The co-administration of 10 mg/kg ABZ for 5 days with this sub-optimal regimen induced synergy in anti-*Wolbachia* efficacy within female *B. pahangi* whereby *Wolbachia* was depleted in all worms to >99%, which was non-inferior to 7-day bi-daily dosing of AWZ1066S with 100 mg/kg (Figure 2B). Further, reducing the regimen of AWZ1066S to 25 mg/kg bi-daily for 5 days in combination with ABZ was inferior to the 7-day bi-daily 100 mg/kg regimen of AWZ1066S in terms of anti-*Wolbachia* efficacy (Dunn's *post-hoc* test *p* < 0.0001), although this combination treatment still mediated a shift in *Wolbachia* depletions compared to the 25 mg/kg 5-day bi-daily AWZ1066S monotherapy (77.9 vs. 0% median depletion).

The impact of *Wolbachia* depletions on embryogenesis and mf production was measured by enumerating the total numbers of mf in gerbils following a 17-week washout of drug treatments. *B. pahangi* mf had accumulated in vehicle control animals to a median level of 1.2 × 10^6^. The 7-day 100 mg/kg bi-daily regimen of AWZ1066S positive control had resulted in a median 99.7% significant depletion in peritoneal mf compared with vehicle controls (Kruskal-Wallis 1-Way-ANOVA *p* < 0.0001, Dunn's *post-hoc* test *p* < 0.001) which were also statistically superior to a median 63% reduction in peritoneal mf mediated by ABZ treatment for 5 days (Figure 2C). All 100–50 mg/kg 5-day AWZ1066S treatments and combinations were statistically non-inferior to the 7-day high dose of AWZ1066S in terms of reducing the numbers of mf to >99%. The impact of combination synergy was, however, observable at the lowest dose of AWZ1066S tested, whereby monotherapy for 5 days two times daily did not significantly lower mf yields compared with vehicle levels and thus was statistically inferior to the 7-day 100 mg/kg dosing (1.5% median reduction, Dunn's *post-hoc* test *p* < 0.0001). Combining this low AWZ1066S dose with ABZ reverted mf depletion levels to, on average, 73.5%, which was non-inferior to high dose efficacy.

Because of the longevity of lymphatic filarial mf (60–100 weeks half-life) (Eberhard, 1986), and because we had commenced treatments after the establishment of fecund infections, residual low levels of mf recorded within treated gerbils may reflect the presence of mf released prior to *Wolbachia* depletions and knock-on block of embryogenesis. Therefore, we also enumerated mf released *ex vivo* from live female *B. pahangi* cultured overnight following their isolation from gerbils treated with AWZ1066S and ABZ combinations (Figure 2D). Vehicle control-treated female worms released a median of 2,600 mf overnight, with a minority of 14/45 worms non-productive in mf release (31%). ABZ treatment reduced the median level of mf released to 300 but with a similar number of unproductive female worms (25/55; 45%). Comparatively, the high-dose 100 mg/kg 7 bi-daily regimen of AWZ1066S had significantly prevented mf release in 83% (15/18) of female worms in culture (Kruskal-Wallis 1-Way-ANOVA *p* < 0.0001, Dunn's *post-hoc* test *p* < 0.001 and *p* < 0.05 vs. vehicle and ABZ treatment groups, respectively). Between 83–95% of *B. pahangi* worms assayed were unproductive in releasing mf in 100–50 mg/kg AWZ1066S 5-day treatment groups, irrespective of ABZ co-dosing. However, when reducing the *in vivo* dose exposure of AWZ1066S to 25 mg/kg bi-daily for 7 days, only a minority of female worms assayed (9/28, 32%) were unproductive in mf release, which was significantly inferior to the mf blocking activity of 7-day 100 mg/kg AWZ1066S (Dunn's *post-hoc* test *p* < 0.01). Co-dosing this inferior regimen of AWZ1066S with ABZ blocked mf production within 64% of female worms in culture (14/22).

There were no significant differences in total adult, female or male worm burdens between groups at 18 weeks post-treatment, and recoveries in vehicle controls were highly variable (median 72 worms per gerbil, range 16–121, Figures 2E–G). However, both 7-day and 5-day 100 mg/kg bi-daily AWZ1066S dosing had resulted in a trend towards lower adult burdens (60.8 and 45.3% median reductions, respectively), whilst gerbils receiving lowered doses of AWZ1066S were more similar to vehicle control levels with the exception of 50 mg/kg combined with ABZ, which resulted in a 37.8% median reduction.

**Figure 3:**
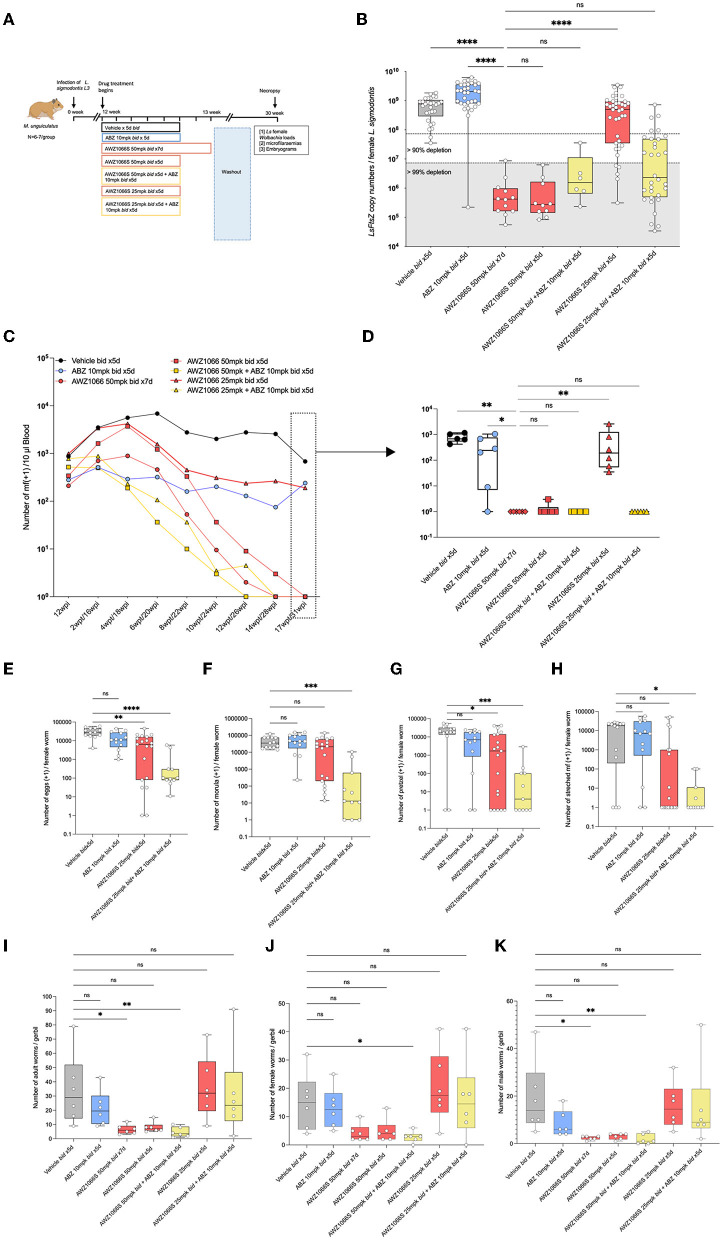
Synergistic depletion of *Wolbachia* in female *L. sigmodontis*, clearance of mf from circulation and adulticidal activity after a low-dose 5-day AWZ1066S and ABZ combination therapy. **(A)** Schematic representation of experimental design. **(B)** *Wolbachia* numbers in individual female *L. sigmodontis* measured by qPCR for the single copy *Wolbachia* gene, ftsz. **(C)** Time course of median microfilaraemias per group following drug treatment. **(D)** Comparison of microfilaraemias at end-point. **(E)** Embryogram enumeration of eggs. **(F)** Early-stage morulae. **(G)** Pretzel-stage coiled mf. **(H)** Stretched mf from uteri of female *L. sigmodontis* at end-point. **(I)** Total numbers, **(J)** female, and **(K)** male *L. sigmodontis* adults at end-point. Box and whiskers indicate min/max, median and interquartile range with individual data overlayed. Significance is indicated ns, *p* < 0.05*, *p* < 0.01**, *p* < 0.001***, and *p* < 0.0001**** calculated by the Kruskal-Wallis test with Dunn's multiple comparisons. Data is derived from *n* = 7–8 gerbils/group from a single experiment. ns, not significant.

We then utilised an *L. sigmodontis* infection model to further assess the impact of co-administration of ABZ with low-dose titrations of AWZ1066S in *Wolbachia* depletion and embryogenesis inhibition. In this rodent-adapted filarial infection model, mf produced from fecund infections migrate from the adult thoracic cavity infection site and establish long-term parasitaemias. We had previously defined that 50 mg/kg AWZ1066S two times daily for 7 days was sufficient to deplete *Wolbachia* >99% and remove mf from circulation in this model (Hong et al., 2019). Following 12 weeks of infection, *M. unguiculatus* gerbils were treated at or below this reference dose of AWZ1066S as a monotherapy or in combination with 10 mg/kg ABZ. The effects of treatment were evaluated following a long washout period of 16 weeks (Figure 3A). The AWZ1066S 50 mg/kg bi-daily 7-day regimen mediated the predicted >99% depletion of *Wolbachia* within *L. sigmodontis* female adults, whereas ABZ monotherapy was not efficacious (Kruskal-Wallis 1-way-ANOVA *p* < 0.0001, Dunn's *post-hoc* test *p* < 0.0001; Figure 3B). Lowering the dose time-frame of 50 mg/kg of AWZ1066S to 5 days was equally >99% efficacious in *Wolbachia* depletions, irrespective of ABZ co-dosing (Figure 3B). However, 25 mg/kg AWZ1066S alone mediated a 35% median reduction in *Wolbachia*, which was inferior to the 7-day 50 mg/kg dosing (Dunn's *post-hoc* test *p* < 0.0001). Yet, the co-administration of ABZ with 25 mg/kg AWZ1066S rescued *Wolbachia* depletion efficacy to a >99% median level (Figure 3B).

From the point of treatment, we assessed peripheral blood *L. sigmodontis* microfilaraemias every 2 weeks. In vehicle controls, peripheral microfilaraemias remained constant over the post-treatment time period, at a median range of between 941 and 785 mf/10μl blood (Figure 3C). The reference 7-day bi-daily 50 mg/kg dose of AWZ1066S gradually lowered mf in the blood and, at 17-week post-treatment, all gerbils were amicrofilaraemic (Kruskal-Wallis 1-way-ANOVA *p* < 0.0001, Dunn's *post-hoc* test *p* < 0.01; Figure 3D). ABZ monotherapy did not deplete mf from peripheral circulation and was inferior to the AWZ1066S 7-day dosing (Dunn's *post-hoc* test *p* < 0.05). Lowering the dose time-frame of 50 mg/kg AWZ1066S to 5 days was equally 100% efficacious in the gradual clearance of mf from circulation, irrespective of ABZ co-dosing. However, lowering the 5-day dosage of AWZ1066S to 25 mg/kg bi-daily was insufficient to mediate clearance of mf from circulation (*p* < 0.01 compared with the 7-day 50 mg/kg dosing; Figure 3D). ABZ co-administration at this dose level resulted in complete efficacy in the gradual removal of mf from the blood of infected gerbils (Figures 3C, D). Embryograms of uterine content in *L. sigmodontis* female worms at 17 weeks post-treatment confirmed that whilst ABZ monotherapy alone had no significant impact in embryogenic stages *in uteri*, co-dosing had significantly augmented the otherwise sub-optimal embryotoxic activity of 7-day bi-daily 25 mg/kg AWZ1066S (Figures 3E–H).

*L. sigmodontis* adult worm burdens were significantly reduced following the AWZ1066S 50 mg/kg regimen dose for 7 days (a 79% median reduction compared with vehicle controls, range 52–83%, Kruskal-Wallis 1-Way-ANOVA *p* = 0.0001, Dunn's *post-hoc* test *p* < 0.05; Figure 3I). Of the reduced 5-day regimens, only the combination of 50 mg/kg AWZ1066S with ABZ mediated a similar, significant 88% reduction in adult worm burden (range 66–97%, Dunn's *post-hoc* test *p* < 0.05; Figure 3I). Both female and male worm burdens were significantly reduced following this 5-day combination drug exposure (Figures 3J, K).

**Figure 4:**
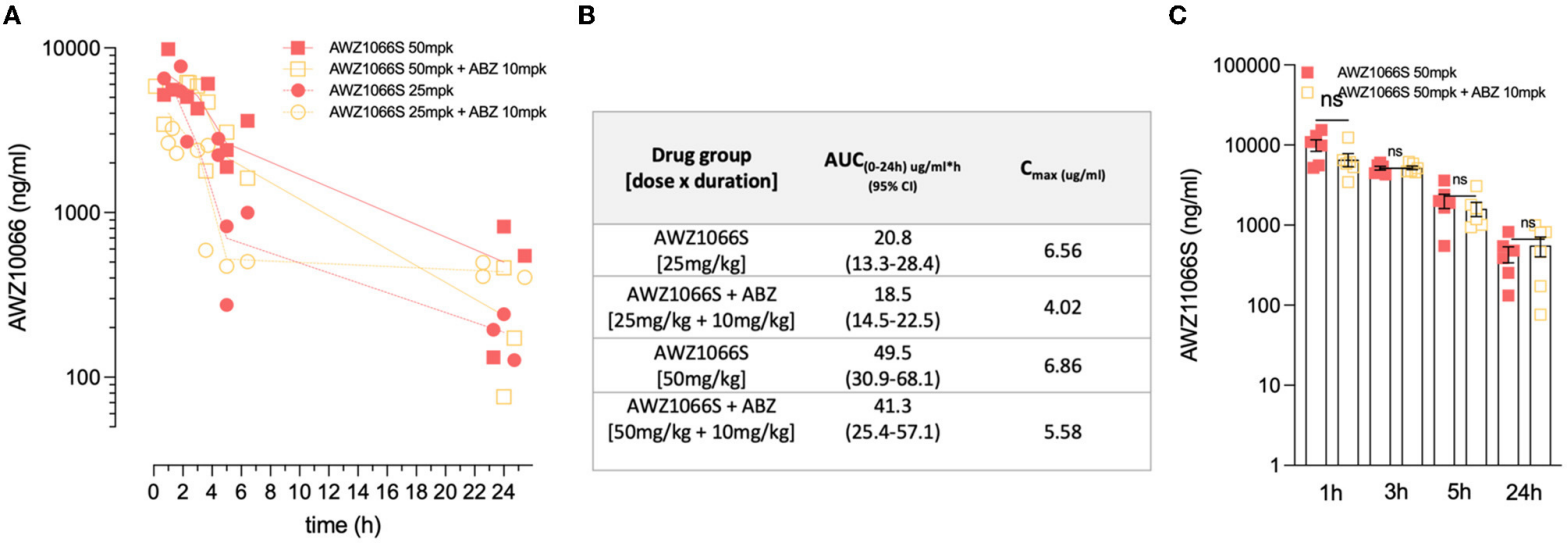
ABZ co-dosing does not significantly alter AWZ1066S drug exposure profiles. **(A)** AWZ1066S blood concentrations. **(B)** Summary PK parameters over 24 h in indicated groups. **(C)** Combined AWZ1066S blood exposure data adjusted to 50 mg/kg (assuming linearity with or without ABZ co-dosing). No significant (ns) differences when compared to Student's *t*-test. Data from individual gerbils and time-points are plotted from groups of 3–6 gerbils. Bars and error bars represent mean ± SEM.

The exposure profiles of AWZ1066S were measured over 24 h post-first dose at 1, 3, 5, or 24 h in groups of 3 *L. sigmodontis-*infected gerbils receiving 50 or 25 mg/kg AWZ1066S with or without ABZ co-administrations (Figure 4A). The area under the curve (AUC_0−24*h*_) of AWZ1066S was dose proportional, and both AUCs and peak plasma levels (C_max_) were similar in gerbils receiving either monotherapy or ABZ combination (Figure 4B). When adjusting to the 50 mg/kg dose and combining data (*n* = 6 gerbils per group), co-dosing of ABZ did not cause any significant alteration in AWZ1066S blood concentration at any time-point measured (Figure 4C), indicating a lack of drug-drug interaction. Combined, these data demonstrate that ABZ can augment the anti-*Wolbachia* activity of sub-optimal, low-dose 5-day exposures of the investigational drug AWZ1066S, with concomitant long-term significant impacts on complete transmission blockade and partial adulticidal activities.

### AWZ1066S-ABZ combination augments *Wolbachia* depletions within uterine and hypodermal chords

**Figure 5:**
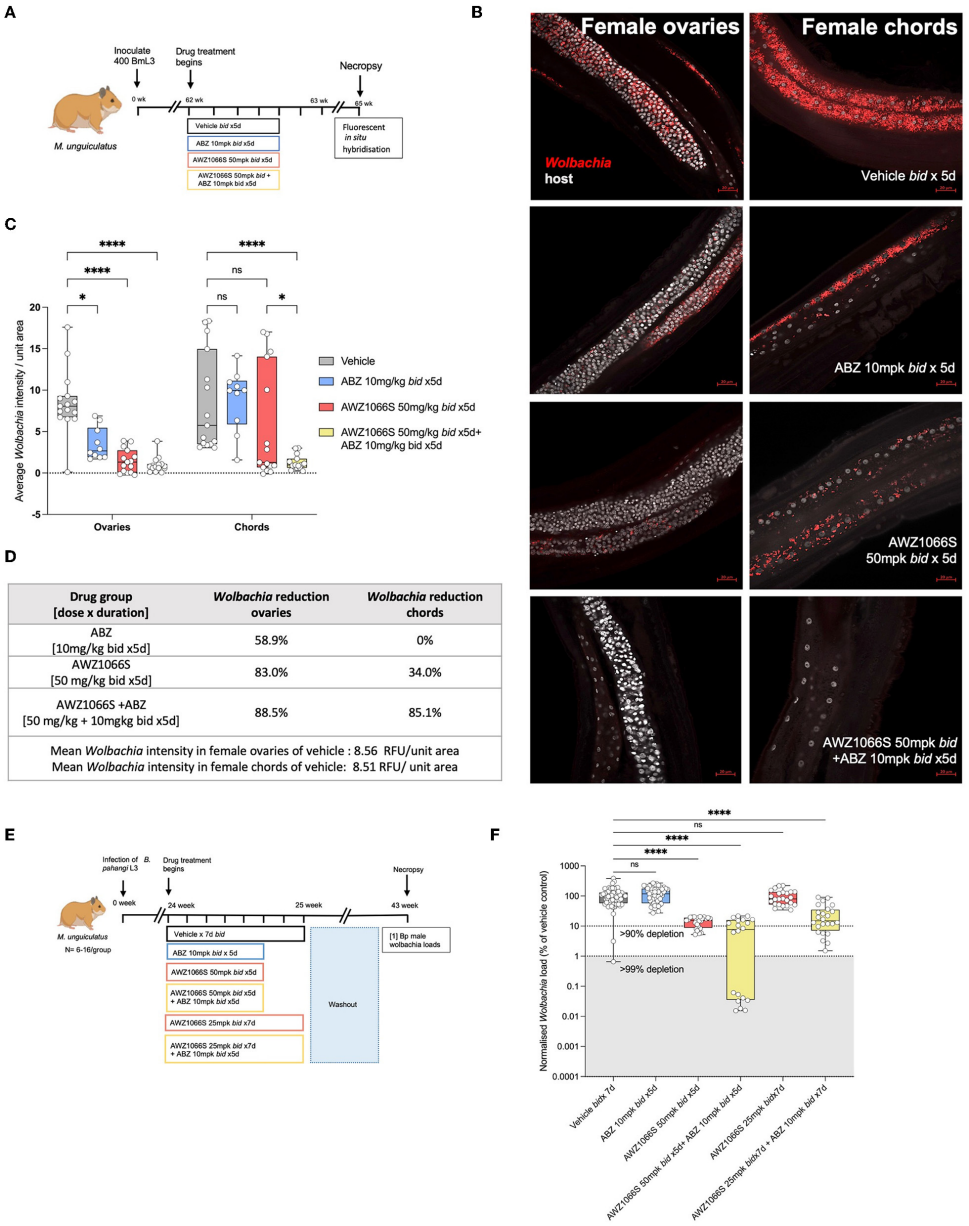
Treatment of AWZ1066S with ABZ synergistically targets both uterine and hypodermal chord *Wolbachia*. **(A)** Schematic representation of experimental design. **(B)** Representative FISH photomicrographs using *Wolbachia* (red) specific probes against 16S rRNA gene. Bars −20 μm. **(C)** Morphometric analysis of FISH signal intensity. Data plotted is *Wolbachia* intensity per field of view from three individual worms combined. Bars represent mean *Wolbachia* intensity ± SEM. Significance is indicated *p* < 0.05*, *p* < 0.0001**** calculated by 2-way-Anova with Šídák's multiple comparisons *post-hoc* test. **(D)** Table summarising *Wolbachia* depletion in ovarian and hypodermal chord populations. **(E)** Experimental design and **(F)** *Wolbachia* loads in male *B. pahangi* following *in vivo* exposure to indicated doses of AWZ1066S ± ABZ. Box and whiskers indicate min/max, median and interquartile range with individual data overlayed. Significance is indicated *p* < 0.0001**** calculated by the Kruskal-Wallis test with Dunn's multiple comparisons. Data is derived from *n* = 7–8 gerbils/group from a single experiment. ns, not significant.

Filarial *Wolbachia* resides in two different tissues in female *B. malayi*—the hypodermal chord syncytium and female germline (Taylor and Hoerauf, 1999; Taylor et al., 1999; Landmann et al., 2010). To understand the initial dynamics of AWZ1066S monotherapy or in combination with ABZ treatment on distinct *Wolbachia* tissue populations, we used a *B. malayi* infection model in *M. unguiculatus* gerbils. Adult parasites were recovered after 2 weeks of drug treatment with either 50 mg/kg *bid* AWZ1066S alone or in combination with 10 mg/kg ABZ for 5 days. Vehicle and 10 mg/kg ABZ control groups were also included (Figure 5A). Using previously established FISH staining with *Wolbachia-*specific 16S rRNA probes (Walker et al., 2021; Marriott et al., 2023), we visualised and quantified *Wolbachia* loads both in hypodermal chords and ovaries in female worms from control and different drug treatment groups. Previous studies have shown that the ovaries have the highest density of germline *Wolbachia* populations (Bakowski et al., 2019) and are also the only tissue site in mature adult worms containing proliferating cells (Foray et al., 2018); thus, they serve as an ideal tissue to compare against hypodermal chords. Via measuring *Wolbachia* fluorescent signal intensity from randomly selected fields of view, AWZ1066S monotherapy resulted in a significant 83% mean depletion in ovaries (2-way-ANOVA *p* < 0.0001, Šídák's multiple comparisons *post-hoc* test *p* < 0.0001 vs. vehicle; Figures 5B–D) whereas only a non-significant average 35% depletion was observed in hypodermal chords compared to the respective tissues in vehicle-treated worms. ABZ alone had a non-significant mean 59% effect on *Wolbachia* levels in ovaries and no impact on *Wolbachia* levels in hypodermal chords. The effect of ABZ combination therapy with AWZ1066S was the augmentation of a significant 88.5% depletion of *Wolbachia* within both hypodermal chord and ovarian populations (*p* < 0.0001, Figures 5B–D). We then examined *Wolbachia* depletion levels in male *B. pahangi* following short-course exposures of AWZ1066S ± ABZ (Figure 5E), whereby effects would be limited to the hypodermis. Whilst ABZ only had no impact on male *Wolbachia* loads, treatment of AWZ1066S at 50 mg/kg bi-daily for 5 days in combination with ABZ reduced the median *Wolbachia* depletion level from 84.8 to 92.3% (both statistically significant vs. vehicle; Kruskal-Wallis 1-way-ANOVA *p* < 0.0001, Dunn's *post-hoc* tests *p* < 0.0001). When further de-escalating the dose of AWZ1066S to 25 mg/kg two times daily, only the combination with ABZ mediated a significant 85.1% median *Wolbachia* depletion (Dunn's *post-hoc* test *p* < 0.0001 vs. vehicle; Figure 5E). Together, these data demonstrate that whilst the germline population is more sensitive to depletion by low-dose, short-course AWZ1066S treatments, ABZ can augment the depletion of nematode *Wolbachia* residing within both germline and hypodermal tissues.

## Discussion

We report a pharmacological synergy between the first-in-class azaquinazoline anti-*Wolbachia* small molecule, AWZ1066S, and anthelmintic benzimidazoles (albendazole, oxfendazole), in targeting *Wolbachia* within filarial worms in multiple rodent infection models. The resulting combination treatment delivers minimal effective exposure durations of 5 days whilst concomitantly decreasing the systemic exposure of AWZ1066S by ~2-fold necessary to achieve at least 90% *Wolbachia* depletion, a clinically determined minimum threshold for delivering slow-cure macrofilaricidal activity in lymphatic filariasis (Johnston et al., 2021).

The data in our study confirms the profound *Wolbachia* depletion from filarial tissues by AWZ1066S. AWZ1066S is a unique narrow-spectrum anti-*Wolbachia* compound with no general antibiotic properties and rapid bactericidal kinetics, achieving near maximal clearance of nematode *Wolbachia in vitro* following exposure of 1 day vs. 6 days required for tetracyclines, rifamycins, and fluoroquinolones (Hong et al., 2019; Johnston et al., 2021). We have previously established that regimens between 100 and 50 mg/kg two times daily for 7 days are efficacious in delivering a threshold >90% anti-*Wolbachia* activity against *B. malayi* immature adults in SCID mice and *L. sigmodontis* in gerbils, respectively, leading to total block of mf production via embryostasis. In addition, we determined that via depletion of *Wolbachia* in the mf-stage, AWZ1066S can also block the development of *Brugia* in the mosquito vector via a deficit in *Wolbachia*, inhibition of mf chitinase production and failure to exsheath in the insect midgut (Quek et al., 2022). Most recently, we have determined 90% anti-*Wolbachia* activity of AWZ1066S against developing larvae of the veterinary filaria, *Dirofilaria immitis*, following 2-day exposures *in vivo*, extending the potential use-case of this new class of anti-*Wolbachia* therapeutic from human to veterinary medicine (Turner et al., 2020; Marriott et al., 2023). In this study, compared with a 100 mg per day human equivalent dose exposure of doxycycline (Sharma et al., 2016), which requires 6 weeks in a SCID model of brugian filariasis to mediate >90% anti-*Wolbachia* activity, we further established the minimum AWZ1066S dose time frame of 5 days which could sustainably deplete *Wolbachia* to a similar or >99% threshold when sufficient bi-daily dose-exposures were applied against mature *B. pahangi* in gerbils (Figures 1, 2). We further corroborated a minimum >90% anti-*Wolbachia* effect in a complementary *L. sigmodontis* gerbil model with a 50 mg/kg 5-day bi-daily regimen. Extending our prior data (Hong et al., 2019), we also confirmed no rebound of *Wolbachia* with these minimum 5-day bi-daily dose AWZ1066S regimens up to 18 weeks post-treatment in both models (Figure 3). These 5-day monotherapeutic doses led to a complete cessation of mf production in 95% of mature fecund female *Brugia pahangi* assayed whilst delivering a gradual and complete clearance of *L. sigmodontis* mf from the circulation, mediated via block of embryogenesis (Figures 2, 3). Importantly, in these long-term models, we could also begin to discern significant macrofilaricidal activity, with reduced adult worm burdens following treatment compared with vehicle controls (Figure 3). Typically, 12 months is a minimum necessary time frame for induction of significant macrofilaricidal activity following the loss of the endosymbiont via 6-week doxycycline therapy in clinical LF trials, judged by loss of ultrasonograph “filarial dance sign” in intra-lymphatic worm nests (Debrah et al., 2006). However, we have previously reported that high-dose rifampicin, when combined with ABZ to augment *Wolbachia* depletions, can begin to affect adult worm burdens in as short as 6 weeks following treatment in a SCID mouse model of brugian filariasis (Aljayyoussi et al., 2017; Turner et al., 2017). The accelerated AWZ1066S-mediated macrofilaricidal activity after 18 weeks might, therefore, reflect the rapid *Wolbachia* kill kinetics of this azaquinazoline agent compared with registered antibiotics.

Our data reinforces that *Wolbachia* is an exquisitely selective drug target whereby it contributes towards multiple fundamental biological processes underpinning the obligatory mutualism with its filarial host. *Wolbachia* is postulated to provide a source of nucleotides and micronutrients (including flavin adenine dinucleotide, haem and riboflavin) to meet the metabolic demands of filariae, particularly in periods of rapid growth, as gene pathways intact in the symbiont are either insufficient or lacking in *Wolbachia* containing filarial worms (Slatko et al., 2010).

Other cell biology processes that *Wolbachia* is implicated in regulating across nematode and insect hosts include oxidative stress, autophagy and apoptosis (Landmann et al., 2011; Voronin et al., 2012; Gill et al., 2014). *Wolbachia* localises in two major tissues within filariae—the hypodermal chord syncytium and the female germline (Slatko et al., 2010; Taylor et al., 2012). A rapid manifestation of antibiotic depletion of endobacteria from the female filarial germline is a widespread apoptosis spreading from *Wolbachia-*containing germline cells to non-Wolbachia-containing somatic tissues of developing embryos and mf (Landmann et al., 2011). More recent evidence from spatiotemporal microscopy studies suggests antibiotic *Wolbachia* removal from the female germline initiates a dysregulation of stem cell proliferation, leading to a significant diminution and disorganisation of the ovary mitotic zone, which precedes widespread apoptosis of embryos leading to sterility (Foray et al., 2018). In comparison, rapid apoptosis is not a tissue-specific feature following depletion of hypodermal *Wolbachia*. It is thus postulated that permanent clearance of the hypodermal *Wolbachia* population leads to a deficit of nutrient sources for prolonged survival in the adult filarial parasitic niche (Slatko et al., 2010; Landmann et al., 2011). An alternate hypothesis, supported by cellular changes in white blood cell composition, localisation and granulocyte-released effector molecules surrounding adult *Onchocerca* post-*Wolbachia* removal, is that endobacterial titres aid the parasite subversion of an otherwise complex, attritional anti-parasite host immune response (Hansen et al., 2011; Tamarozzi et al., 2016). This spatially and temporally segregated impact of *Wolbachia* removal from distinct filarial tissue sites may explain why lowered-dose doxycycline exposures in LF clinical trials lead to an irreversible block of embryogenesis but not significant macrofilaricidal activity (Turner et al., 2006). Consistent with a hypothesis that reproductive tissues are a more sensitive site for drug-mediated *Wolbachia* clearance, in this study, we observed that sub-optimal dosing of AWZ1066S monotherapy mediated more consistent *Wolbachia* depletions in ovaries than in those of the lateral chords (Figure 5). Contrasting depletion levels by lowered dose exposures of anti-*Wolbachia* drugs in different filarial tissues may reflect effect site pharmacokinetics (i.e., local drug penetration or drug detoxification) or that the *Wolbachia* residing in the reproductive tissues are intrinsically more sensitive to drug activity, potentially via virtue of higher division rate and metabolic activity. Recent dual RNAseq analysis of *Wolbachia* gene transcription in the hypodermal vs. the ovarian population within *B. malayi* indicates a relatively harsher environment in the former location, reflected by upregulation of bacterial stress response proteins, which may impinge on endobacterial growth (Chevignon et al., 2021).

Upon combining humanised dose exposures of ABZ equivalent to standard 400 mg dosing (Turner et al., 2017) or matching doses of the veterinary OXF, which has higher systemic exposure and is being repurposed for human helminth indications (Lanusse et al., 1995; Gokbulut et al., 2007; Bach et al., 2020; Ehrens et al., 2022; Risch et al., 2023), we could demonstrate a pharmacological synergy of otherwise sub-optimal doses of AWZ1066S given alone for 5 days. The impact of ABZ “rescue” meant these otherwise ~2-fold sub-optimal 5-day doses could recapitulate profound sterilising and partial macrofilaricidal activities in the rodent models and washout periods tested. This indicates synergy is likely consistent across the benzimidazole anthelmintic class. The classical mode of action of benzimidazole drugs, including the active metabolite of ABZ (ABZ-sulphoxide) and OXF, is via capping the alpha β-tubulin subunit to prevent microtubule polymerisation in helminth parasites (Oxberry et al., 2001). Benzimidazole-mediated β-tubulin polymerisation inhibition results in two major deleterious consequences—disruption of cell proliferation leading to apoptosis (Zhang et al., 2017) and defective uptake and transport of energy stores leading to parasite starvation (Lacey, 1990; Keiser and Utzinger, 2010; Chai et al., 2021). In tissue-dwelling filarial nematodes, probably related to low systemic exposures of ABZ compared with gut-dwelling helminths, drug effects are typically transient and non-lethal to adult filariae but lead to temporary disruption of embryogenesis in female adult worms (Cardenas et al., 2010), which is manifest by a gradual, partial reduction in mf in circulation (Awadzi et al., 1991; Klion et al., 1993). The mechanism by which ABZ or OXF can synergise anti-*Wolbachia* drug efficacies with AWZ1066S or other anti-*Wolbachia* agents remains to be resolved. In this study, we found that human equivalent, physiologically relevant dosing of ABZ up to 5 days as monotherapy had no long-term effect on *Wolbachia* abundance *per se* in three different filarial infection models (assayed between 6 and 18 weeks following exposure). Intracellular bacteria are known to utilise the host cytoskeleton system for inter- and intracellular locomotion. For instance, in *Drosophila* cells, *Wolbachia* can also undergo horizontal cell-to-cell transfer (White et al., 2017). Intracellularly, *Wolbachia* resides in host Golgi-related vesicles (Cho et al., 2011), and microtubules play a crucial role in the formation, maintenance, and intracellular locomotion of these vesicles (Cole et al., 1996; Wu et al., 2016). Hence, disruption of microtubule structure by benzimidazole drugs may limit the division and spread of residual surviving *Wolbachia* within the host cell post-removal of AWZ1066S and/or might induce a more bacteriostatic environment augmenting AWZ1066S targeting during dual exposures of drugs.

A further related hypothesis we examined was that synergy in *Wolbachia* depletion exerted by ABZ was targeted specifically to prevent germline cell proliferation and inhibit residual surviving *Wolbachia* spread between germline cells post-drug removal. Whilst long-term, ABZ monotherapy did not adversely affect total endobacterial titres, at 2 weeks post-exposure, we could discern a partial reduction in ovarian *Wolbachia* by microscopy analysis, suggesting a temporary tissue-specific impact of ABZ. Our theory of synergy operating exclusively at the level of the germline was initially corroborated by prior observations that reduced dose exposures of minocycline and rifampicin could lead to enhanced endobacterial depletions in female but not male worms, enumerated from whole worms by PCR (Turner et al., 2017). However, in our spatial FISH microscopy studies reported here, we could resolve significant synergism in both uterine and hypodermal populations of female *B. malayi* with AWZ1066S + ABZ combinations, and we could also discern that AWZ1066S + ABZ could mediate a long-term synergistic depletion of the hypodermal population in male *B. pahangi* (Figure 5). Thus, we confirm ABZ-mediated synergistic hypodermal *Wolbachia* depletion is also demonstrable, which may depend on exposure level, exposure time-frame, drug physiochemical properties aiding penetration and inherent kill-kinetic of the anti-*Wolbachia* agent being combined.

In prior work, we first defined a synergy between registered antibiotics (minocycline or rifampicin) and ABZ at the level of anti-*Wolbachia* efficacy, as well as mf production and accelerated curative efficacy (Turner et al., 2017). Clinically, it has also been demonstrated that ABZ-doxycycline combinations can reduce the dose time frame (from 4 to 3 weeks) for effective *Wolbachia* clearance and embryostatic activity in onchocerciasis (Klarmann-Schulz et al., 2017). Our data herein demonstrates that synergy is also operable when combined with a novel azaquinazoline anti-*Wolbachia* class of drug, and thus benzimidazoles may be a universal synergist that can be applied with new bespoke anti-*Wolbachia* compounds in development (Clare et al., 2019; Johnston et al., 2021) or repurposed antibiotics to lower doses and reduce total exposure periods in line with challenging 7-day dosing requirements for the treatment of LF and onchocerciasis. Beyond human medicine, combinations of azaquinazoline candidates or other rapid-acting novel anti-*Wolbachia* agents with registered benzimidazole drugs may provide new therapeutic options for curing veterinary/zoonotic infections such as *B. malayi* and *D. immitis* infections of cats and dogs.

## Data availability statement

The original contributions presented in the study are included in the article/Supplementary material, further inquiries can be directed to the corresponding author.

## Ethics statement

The animal study was approved by *B. malayi* infection procedures in mice and gerbils were approved by the Animal Welfare and Ethics Review Boards (AWERB) of Liverpool School of Tropical Medicine and University of Liverpool and undertaken in accordance with UK home office licencing approval. *B. pahangi* infection procedures in gerbils were approved by the University of California, San Francisco Institutional Animal Care and Use Committee (IACUC) (approvals: AN109629-03 and AN173847-02) and adhered to the guidelines set forth in the NIH guide for the care and use of laboratory animals and the USDA animal care policies. *L. sigmodontis* study procedures were performed according to the European union animal welfare guidelines and the state office for nature, environment and consumer protection, Cologne, Germany (AZ 84-02.04.2015.A507). The study was conducted in accordance with the local legislation and institutional requirements.

## Author contributions

SH: Formal analysis, Investigation, Methodology, Visualisation, Writing—original draft, Writing—review & editing. AM: Formal analysis, Investigation, Methodology, Writing—review & editing. NP: Formal analysis, Investigation, Methodology, Writing—review & editing. AS: Formal analysis, Investigation, Methodology, Writing—review & editing. CB: Formal analysis, Investigation, Methodology, Writing—review & editing. EG: Formal analysis, Investigation, Methodology, Writing—review & editing. IV: Formal analysis, Investigation, Methodology, Writing—review & editing. MK: Formal analysis, Investigation, Methodology, Writing—review & editing. AE: Formal analysis, Investigation, Methodology, Writing—review & editing. SL: Formal analysis, Investigation, Methodology, Writing—review & editing. DV: Formal analysis, Investigation, Methodology, Visualisation, Writing—review & editing. NT: Formal analysis, Investigation, Methodology, Writing—review & editing. AH: Conceptualisation, Formal analysis, Funding acquisition, Supervision, Writing—review & editing. MH: Conceptualisation, Formal analysis, Funding acquisition, Supervision, Writing—review & editing. JS: Conceptualisation, Formal analysis, Funding acquisition, Supervision, Writing—review & editing. GA: Formal analysis, Investigation, Methodology, Writing—review & editing. FG: Conceptualisation, Funding acquisition, Resources, Supervision, Writing—review & editing. JD: Methodology, Writing—review & editing. DH: Conceptualisation, Formal analysis, Funding acquisition, Investigation, Methodology, Supervision, Writing—review & editing. PO'N: Conceptualisation, Funding acquisition, Supervision, Writing—review & editing. SW: Conceptualisation, Funding acquisition, Supervision, Writing—review & editing. MT: Conceptualisation, Funding acquisition, Supervision, Writing—review & editing. JT: Conceptualisation, Formal analysis, Funding acquisition, Methodology, Supervision, Visualisation, Writing—original draft, Writing—review & editing.
