# Supplementary material for: Combinations of the azaquinazoline anti-Wolbachia agent, AWZ1066S, with benzimidazole anthelmintics synergise to mediate sub-seven-day sterilising and curative efficacies in experimental models of filariasis
Source: Front Microbiol. 2024 Feb 1;15:1346068. doi: 10.3389/fmicb.2024.1346068 (PMC10867176; doi:10.3389/fmicb.2024.1346068)
Supplement: Supplementary file 1 [file Data_Sheet_1.docx]

**Combinations of the azaquinazoline anti-*Wolbachia* agent, AWZ1066S, with benzimidazole anthelmintics synergise to mediate sub-seven day sterilising and curative efficacies in experimental models of filariasis**

**Suplementary data**

**Fig S1: Comparative pharmacokinetic bioanalysis of albendazole (ABZ) in CB.17 SCID mice and Mongolian gerbils (*M. unguiculatus*).**

Pharmacokinetic studies were conducted to determine the equivalent drug exposure in Mongolian gerbils compared to CB.17 SCID mice after the oral administration of ABZ monotherapy. ABZ was dissolved in standard suspension vehicle (0.5% carboxymethyl cellulose, 0.5% benzyl alcohol, 0.4% Tween 80, 0.9% NaCl) for dosing. Bioanalysis to quantify the ABZ-sulfoxide, the active metabolite of ABZ, in CB.17 SCID mice blood after 5mg/kg ABZ dosing was done as described previously [1]. Bioanalysis of ABZ-sulfoxide levels in Mongolian gerbils after 10mg/kg ABZ doseing was done at WuXi Apptech (Couvet, Switzerland) as described in the materials and methods. As previously reported [1], 5mg/kg ABZ dosing in CB.17 SCID mice resulted average drug exposure of 7154 ± 946 ng.h/mL and apparent drug clearance (CL/F) was 0.41 ± 0.24 L/kg. Comparable drug exposure and clearance was observed with 10mg/kg ABZ dosing in Mongolian gerbils (AUC= 7695 ± 1673 ng.h/mL; CL/F= 1.07 ± 0.24 L/kg) (Fig S1A and S1B). No significant differences was observed between the treatments when compared by Student’s T test.


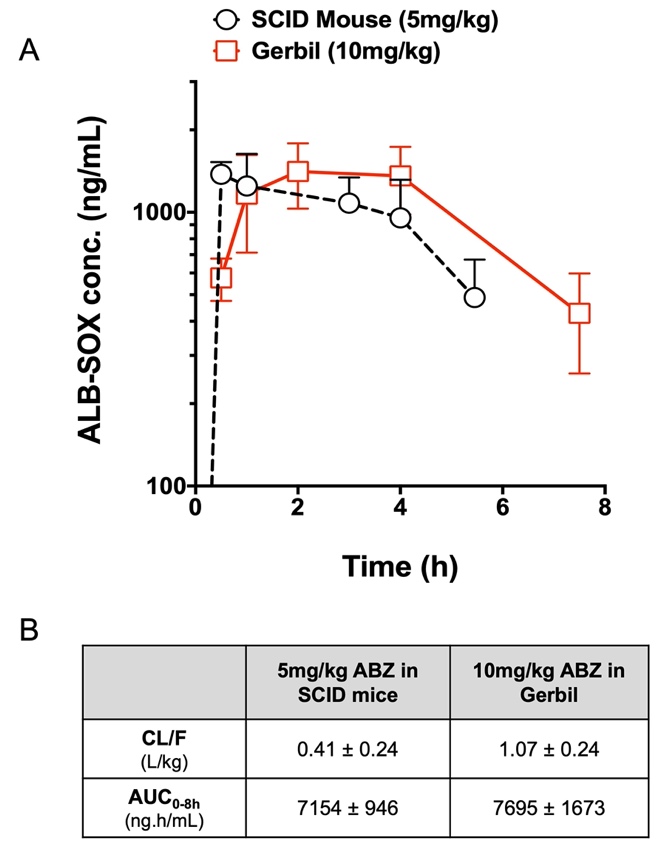


**Table S1: *B. malayi* adult parasites and mf loads post-treatment with AWZ1066S, ABZ, OXF, AWZ1066S+ABZ or AWZ1066S+OXF combination therapy in CB.17 SCID mice.**

*B. malayi* total adult worm burden between vehicle and drug treatment groups was significantly different when analysed by Kruskal-Wallis 1-way-ANOVA (*p* value=0.037) but post-hoc Dunn’s test showed worm burden was not significantly different in any drug treated groups compared to vehicle group (*p*> 0.05). Total peritoneal mf load was significantly different between the groups (Kruskal-Wallis 1-way-ANOVA, *p* value <0.0001). Post-hoc Dunn’s test showed mf levels were significantly different in all other treatment groups (*p*< 0.05) compared to vehicle group except in ABZ 5mpk bid x5d and AWZ1066S 100mpk bid x5d groups.

| **Treatment groups** | **Number of mice (n)** | **Total adult worm burden** | **Median worm burden [min-max]** | **Average worm burgen**  **[±SD, Anova (p)]** | **Median mf load**  **[min-max]** | **Average mf load**  **[SD, Anova (p)]** |
| --- | --- | --- | --- | --- | --- | --- |
| Vehicle | 22 | 202 | 3.5 [0-60] | 9.18 [±15) | 43000  [0-908333] | 115304  [± 222082] |
| DOX 25mpk *bid* x42d | 9 | 62 | 7 [0-12] | 6.88  [±3.8, *p*> 0.05] | 0  [0-640] | 71  [± 213, *p*=0.002] |
| ABZ 5mpk *bid* x5d | 15 | 113 | 7 [0-25] | 7.5  [±7,  *p*> 0.05] | 2333  [0-76666] | 9547  [± 19246, *p*>0.05] |
| 5mpk OXF 5d bid | 5 | 1 | 0 [0-1] | 0.2  [±0.4, *p*> 0.05] | 0  [0-0] | 0  [± 0, *p*=0.01] |
| AWZ1066S 100mpk *bid* x5d | 19 | 116 | 3 [0-22] | 6.1  [±6.6, p>0.05] | 400  [0-108333] | 12124  [± 26824, *p*>0.05] |
| AWZ1066S 100mpk + ABZ 5mpk *bid* x5d | 10 | 90 | 8.5 [0-18] | 9  [±7, *p*> 0.05] | 0  [0-0] | 0  [± 0, *p*<0.0001] |
| AWZ1066S 100mpk + OXF 5mpk bid x5d | 5 | 14 | 0 [0-10] | 2.8  [±4.3, *p*> 0.05] | 0  [0-0] | 0  [± 0, *p*=0.01] |
| AWZ1066S 150mpk *bid* x5d | 5 | 6 | 0 [0-6] | 1.2  [±2.6, *p*> 0.05] | 0  [0-0] | 0  [± 0, *p*=0.0003] |
| AWZ1066S 150 mpk + ABZ 5mpk *bid* x5d | 4 | 38 | 9 [4-16] | 9.5  [±5.5, *p*> 0.05] | 0  [0-0] | 0  [± 0, *p*=0.01] |
| AWZ1066S 100mpk bid x7d | 5 | 30 | 5 [0-12] | 6  [±5, *p*> 0.05] | 0  [0-0] | 0  [± 0, *p*=0.01] |

**Table S2: *B. pahangi* parasites loads post-treatment with AWZ1066S, ABZ or AWZ1066S+ABZ in gerbils**

*B. pahangi* adult worm burden between vehicle and drug treatment groups was not significantly different when analysed by Kruskal-Wallis 1-way-ANOVA (*p* value: total worm burden-0.807; female worm burden-0.587; male worm burden-0.820). Total mf numbers in peritoneal wash were significantly different between the groups (Kruskal-Wallis 1-way-ANOVA. *p* value = 0.0006). Post-hoc Dunn’s test showed mf levels were significantly reduced in 100 mpk AWZ1066S *bid* x7d, 100 mpk AWZ1066S *bid* x5d*,* and 50 mpk AWZ1066S+10 mpk ABZ *bid* x5d treatment groups compared to vehicle group (*p*<0.05).

| **Treatment group** | **Gerbil ID** | **Total females** | **Total males** | **Total adult worms** | **Total mf in 100 mL peritoneal wash** |
| --- | --- | --- | --- | --- | --- |
| Vehicle *bid* x 5d | 1-1 | 19 | 18 | 37 | 150000 |
|  | 1-2 | 14 | 25 | 39 | 5900000 |
|  | 1-3 | 11 | 14 | 25 | 20000 |
|  | 1-4 | 2 | 14 | 16 | 800000 |
|  | 2-1 | 37 | 41 | 78 | 3400000 |
|  | 2-2 | 33 | 32 | 65 | 13700000 |
|  | 2-3 | 62 | 38 | 100 | 16200000 |
|  | 2-4 | 32 | 39 | 71 | 1600000 |
|  | 20-1 | 36 | 21 | 57 | 4100000 |
|  | 20-2 | 32 | 45 | 77 | 4400000 |
|  | 20-3 | 58 | 46 | 104 | 2900000 |
|  | 20-4 | 33 | 44 | 77 | 1300000 |
|  | 20-5 | 39 | 55 | 94 | 1100000 |
|  | 20-6 | 38 | 36 | 74 | 400000 |
|  | 20-7 | 73 | 48 | 121 | 6700000 |
|  | 20-8 | 51 | 48 | 99 | 2200000 |
|  | Median | 34.5 | 38.5 | 75.5 | 1200000 |
|  | Average | 35.625 | 35.25 | 70.875 | 1704813 |
|  | SD | 18.92 | 13.10 | 29.5 | 1929853 |
| 25 mpk AWZ1066S x 7d *bid* | 16-1 | 27 | 27 | 54 | 2100000 |
|  | 16-2 | 55 | 46 | 101 | 2300000 |
|  | 16-3 | 43 | 45 | 88 | 1600000 |
|  | 16-4 | 44 | 55 | 99 | 2300000 |
|  | 17-1 | 33 | 37 | 70 | 900000 |
|  | 17-2 | 16 | 36 | 52 | 630000 |
|  | 17-3 | 25 | 22 | 47 | 1500000 |
|  | 17-4 | 45 | 47 | 92 | 2100000 |
|  | Median | 38 | 41 | 79 | 1850000 |
|  | Average | 36 | 39.3 | 75.3 | 1678750 |
|  | SD | 12.9 | 11.0 | 22.3 | 640456 |
|  | *p* | ns | ns | ns | ns |
| 25 mpk AWZ1066S x 7d *bid* + 10 mpk Albendazole x 5d *bid* | 18-1 | 14 | 24 | 38 | 300000 |
|  | 18-2 | 46 | 45 | 91 | 2100000 |
|  | 18-3 | 23 | 41 | 64 | 1300 |
|  | 18-4 | 22 | 45 | 67 | 1 |
|  | 19-1 | 20 | 31 | 51 | 120000 |
|  | 19-2 | 27 | 38 | 65 | 110000 |
|  | 19-3 | 53 | 50 | 103 | 900000 |
|  | 19-4 | 19 | 53 | 72 | 75000 |
|  | Median | 22.5 | 43 | 66 | 115000 |
|  | Average | 28 | 40.8 | 68.8 | 450787 |
|  | SD | 13.8 | 9.6 | 20.6 | 728643 |
|  | *p* | ns | ns | ns | ns |
| 10 mpk Albendazole x 5d *bid* | 4-1 | 19 | 38 | 57 | 2400000 |
|  | 4-2 | 36 | 39 | 75 | 4200000 |
|  | 4-3 | 38 | 66 | 104 | 2900000 |
|  | 4-4 | 34 | 41 | 75 | 4000000 |
|  | 5-1 | 10 | 18 | 28 | 3200000 |
|  | 5-2 | 3 | 2 | 5 | 20000 |
|  | 5-3 | 6 | 17 | 23 | 1900000 |
|  | 5-4 | 48 | 57 | 105 | 3000000 |
|  | 21-1 | 43 | 25 | 68 | 3000000 |
|  | 21-2 | 56 | 59 | 115 | 23000 |
|  | 21-3 | 61 | 53 | 114 | 2700000 |
|  | 21-4 | 27 | 51 | 78 | 900000 |
|  | 21-5 | 4 | 14 | 18 | 18000 |
|  | 21-6 | 15 | 28 | 43 | 15000 |
|  | Median | 30.5 | 38.5 | 71.5 | 295001 |
|  | Average | 28.57 | 36.28 | 64.85 | 629857 |
|  | SD | 19.52 | 19.47 | 37.09 | 970867 |
|  | *p* | ns | ns | ns | ns |
| 50 mpk AWZ1066S x 5d *bid* | 6-1 | 53 | 37 | 90 | 40000 |
|  | 6-2 | 15 | 18 | 33 | 10000 |
|  | 6-3 | 67 | 56 | 123 | 200000 |
|  | 6-4 | 46 | 42 | 88 | 400000 |
|  | 7-1 | 34 | 64 | 98 | 50000 |
|  | 7-2 | 6 | 20 | 26 | 40000 |
|  | Median | 40 | 40 | 89 | 45000 |
|  | Average | 37 | 40 | 76 | 123333 |
|  | SD | 23 | 19 | 38 | 151349 |
|  | *p* | ns | ns | ns | ns |
| 50 mpk AWZ1066S + 10 mpk Albendazole x 5d *bid* | 8-1 | 47 | 58 | 105 | 30000 |
|  | 8-2 | 17 | 26 | 43 | 20000 |
|  | 8-3 | 17 | 24 | 41 | 40000 |
|  | 8-4 | 10 | 48 | 58 | 40000 |
|  | 9-1 | 3 | 9 | 12 | 0 |
|  | 9-2 | 21 | 28 | 49 | 30000 |
|  | Median | 17.0 | 27.0 | 46.0 | 30000.0 |
|  | Average | 19.2 | 32.2 | 51.3 | 26666.7 |
|  | SD | 15.1 | 17.8 | 30.5 | 15055.5 |
|  | *p* | ns | ns | ns | 0.01 |
| 100 mpk AWZ1066S x 5d *bid* | 10-1 | 63 | 44 | 107 | 200000 |
|  | 10-2 | 16 | 23 | 39 | 0 |
|  | 10-3 | 2 | 0 | 2 | 0 |
|  | 10-4 | 25 | 37 | 62 | 10000 |
|  | 11-1 | 6 | 29 | 35 | 50000 |
|  | 11-2 | 18 | 24 | 42 | 40000 |
|  | Median | 17 | 27 | 41 | 25000 |
|  | Average | 22 | 26 | 48 | 50000 |
|  | SD | 22 | 15 | 35 | 76420 |
|  | *p* | ns | ns | ns | 0.02 |
| 100 mpk AWZ1066S x 7d *bid* | 14-1 | 9 | 20 | 29 | 0 |
|  | 14-2 | 5 | 24 | 29 | 0 |
|  | 14-3 | 11 | 17 | 28 | 20000 |
|  | 14-4 | 13 | 36 | 49 | 60000 |
|  | 15-1 | 8 | 19 | 27 | 230000 |
|  | 15-2 | 40 | 53 | 93 | 0 |
|  | 15-3 | 15 | 20 | 35 | 0 |
|  | Median | 11 | 20 | 29 | 0 |
|  | Average | 14 | 27 | 41 | 44286 |
|  | SD | 12 | 13 | 24 | 84825 |
|  | *p* | ns | ns | ns | 0.004 |

**Table S3: *L. sigmodontis* parasites loads post-treatment with AWZ1066S, ABZ or AWZ1066S+ABZ in gerbils**

*L. sigmodontis* adult worm burden between vehicle and drug treatment groups was significantly different when analyzed by Kruskal-Wallis 1-way-ANOVA (*p* value: total worm burden-0.0001; female worm burden-0.0016; male worm burden- <0.0001). Post-hoc Dunn’s test showed adult worm burden was significantly reduced in 50mpk AWZ1066S *bid* x7d (*p*= 0.046), 50mpk AWZ1066S *bid* x5d + 10 mpk ABZ x5d (*p*=0.0082) compared to vehicle group. Male worm burden was significantly reduced in 50mpk AWZ1066S *bid* x7d, 50mpk AWZ1066S *bid* x5d + 10 mpk ABZ x5d, 100mpk AWZ1066S *bid* x7d and 100mpk AWZ1066S *bid* x5d + 10 mpk ABZ x5d treatment groups compared to vehicle group (*p*<0.05). Female worm burden was significantly reduced in 50mpk AWZ1066S *bid* x5d + 10 mpk ABZ x5d (*p*<0.05) treatment group comped to vehicle. Total thoracic cavity mf numbers were not significantly different in the treatment groups compared to control group (Kruskal-Wallis 1-way-ANOVA, *p* value = 0.116).

| **Treatment** | **Gerbil ID** | **Total females** | **Total males** | **Total adult worms** | **Peritoneal mf SPLASH® II LIPIDOMIX® Mass Spec Standard**  **/ml** |
| --- | --- | --- | --- | --- | --- |
| Vehicle *bid* x 5d | 1  2  3  4  5  6  2  3  4  5  6 | 17 | 18 | 35 |  |
|  | 2 | 4 | 5 | 9 | 95751 |
|  | 3 | 6 | 10 | 16 | 5751 |
|  | 4 | 32 | 47 | 79 | 309001 |
|  | 5 | 19 | 24 | 43 | 132501 |
|  | 6 | 13 | 10 | 23 | 49251 |
|  | Median | 15.00 | 14.00 | 29.00 | 95751 |
|  | Average | 15.17 | 19.00 | 34.17 | 118451 |
|  | SD | 10.15 | 15.28 | 25.21 | 116738 |
| ABZ 10mg/kg *bid* x5d | 52 | 5 | 4 | 9 | 89251 |
|  | 53 | 25 | 18 | 43 | 7251 |
|  | 54 | 7 | 4 | 11 | 7501 |
|  | 62 | 16 | 6 | 22 | 60501 |
|  | 64 | 11 | 6 | 17 | 2751 |
|  | 65 | 14 | 12 | 26 | 35501 |
|  | Median | 12.5 | 6 | 19.5 | 21501 |
|  | Average | 13 | 8.33 | 21.33 | 33792 |
|  | SD | 7.18 | 5.57 | 12.4 | 35074 |
|  | *p* | ns | ns | ns | ns |
| AWZ1066S 25mg/kg *bid* x5d | 7 | 28 | 20 | 48 | 201001 |
|  | 8 | 19 | 11 | 30 | 11251 |
|  | 9 | 16 | 18 | 34 | 72251 |
|  | 10 | 41 | 32 | 73 | 171501 |
|  | 11 | 4 | 5 | 9 | 92501 |
|  | 12 | 14 | 9 | 23 | 1126 |
|  | Median | 17.5 | 14.5 | 32 | 82376 |
|  | Average | 20.33 | 15.83 | 36.17 | 91605 |
|  | SD | 12.75 | 9.7 | 22.14 | 81693 |
|  | *p* | ns | ns | ns | ns |
| AWZ1066S 25mg/kg bid x5d + ABZ 10mg/kg bid x5d | 13 | 41 | 50 | 91 | 437001 |
|  | 14 | 0 | 2 | 2 | 3001 |
|  | 15 | 11 | 10 | 21 | 2501 |
|  | 16 | 18 | 14 | 32 | 111 |
|  | 17 | 18 | 8 | 26 | 11001 |
|  | 18 | 8 | 8 | 16 | 6501 |
|  | Median | 14.50 | 9.00 | 23.50 | 4751 |
|  | Average | 16.00 | 15.33 | 31.33 | 76686 |
|  | SD | 13.99 | 17.42 | 30.96 | 176558 |
|  | *p* | ns | ns | ns | ns |
| AWZ1066S 50mg/kg bid x5d | 37 | 4 | 4 | 8 | 18251 |
|  | 38 | 4 | 4 | 8 | 2291 |
|  | 39 | 5 | 1 | 6 | 326 |
|  | 40 | 2 | 4 | 6 | 2501 |
|  | 41 | 2 | 3 | 5 | 8501 |
|  | 42 | 13 | 2 | 15 | 12751 |
|  | Median | 4 | 3.5 | 7 | 5501 |
|  | Average | 5 | 3 | 8 | 7436 |
|  | SD | 4.1 | 1.26 | 3.63 | 7038 |
|  | *p* | ns | ns | ns | ns |
| AWZ1066S 50mg/kg bid x5d + ABZ 10mg/kg bid x5d | 43 | 0 | 1 | 1 | 3501 |
|  | 44 | 3 | 5 | 8 | 6251 |
|  | 45 | 3 | 1 | 4 | 3501 |
|  | 46 | 3 | 0 | 3 | 6 |
|  | 47 | 2 | 0 | 2 | 11251 |
|  | 48 | 6 | 4 | 10 | 3751 |
|  | Median | 3 | 1 | 3.5 | 3626 |
|  | Average | 2.83 | 1.83 | 4.67 | 4710 |
|  | SD | 1.94 | 2.14 | 3.56 | 3771 |
|  | *p* | 0.04 | 0.007 | 0.008 | ns |
| AWZ1066S 50mg/kg bid x7d | 19 | 10 | 2 | 12 | 146501 |
|  | 20 | 2 | 1 | 3 | 1 |
|  | 21 | 4 | 3 | 7 | 87001 |
|  | 24 | 2 | 2 | 4 | 12751 |
|  | 50 | 2 | 3 | 5 | 6751 |
|  | 51 | 5 | 3 | 8 | 2751 |
|  | Median | 3 | 2.5 | 6 | 9751 |
|  | Average | 4.17 | 2.33 | 6.5 | 42626 |
|  | SD | 3.13 | 0.82 | 3.27 | 60573 |
|  | *p* | ns | 0.02 | 0.04 | ns |

**Table S4: Peripheral microfilaria numbers post-treatment with AWZ1066S alone or in combination with ABZ in gerbils**

Table summarises the total number of circulating mf (+1) / 20ul blood in gerbils dosed with Vehicle, DOX 40 mpk *bid* x14d, different concentration of AWZ1066S alone or in combination with 10 mpk ABZ *bid* x5d.

|  | **12wpi** | **2wpt/16wpi** | **4wpt/18wpi** | **6wpt/20wpi** | **8wpt/22wpi** | **10wpt/24wpi** | **12wpt/26wpi** | **14wpt/28wpi** | **17wpt/31wpi** |
| --- | --- | --- | --- | --- | --- | --- | --- | --- | --- |
| **Vehicle** | 941 | 3641 | 4801 | 5901 | 1341 | 2181 | 3101 | 3281 |  |
|  | 321 | 1401 | 2341 | 2041 | 1041 | 2301 | 2361 | 1701 | 681 |
|  | 421 | 1941 | 3861 | 4501 | 1601 | 1861 | 1681 | 301 | 421 |
|  | 1721 | 5381 | 12267 | 15821 | 5481 | 7961 | 8101 | 2381 | 1021 |
|  | 801 | 3361 | 6441 | 37321 | 5261 | 1441 | 3901 | 3181 | 1161 |
|  | 1441 | 4081 | 12241 | 7841 | 3921 | 1061 | 2421 | 2761 | 641 |
| **Average** | 941 | 3301 | 6992 | 12237 | 3107 | 2801 | 3594 | 2267 | 785 |
| **SD** | 553 | 1450 | 4287 | 13155 | 2029 | 2569 | 2332 | 1123 | 300 |
| **AWZ1066 25 mpk bid x5d** | 1081 | 3161 | 2641 | 1101 | 561 | 561 | 3601 | 3421 | 2581 |
|  | 1341 | 3641 | 3721 | 1381 | 341 | 401 | 201 | 290 | 261 |
|  | 901 | 2101 | 3881 | 2981 | 1101 | 79 | 275 | 455 | 821 |
|  | 2701 | 6321 | 11861 | 4461 | 981 | 221 | 15 | 1 | 35 |
|  | 281 | 1281 | 4521 | 421 | 41 | 6 | 1 | 31 | 59 |
|  | 801 | 4501 | 5401 | 1741 | 261 | 401 | 361 | 238 | 121 |
| **Average** | 1184.33 | 3501.00 | 5337.67 | 2014.33 | 547.67 | 278.17 | 742.33 | 739.33 | 646.33 |
| **SD** | 822.04 | 1787.51 | 3323.62 | 1467.61 | 418.51 | 213.16 | 1407.65 | 1324.56 | 991.62 |
| **p (ANOVA)** | 1.00 | 1.00 | 0.42 | <0.0001 | 0.06 | 0.06 | 0.03 | 0.51 | 1.00 |
| **AWZ1066 25 mpk bid + ABZ 10 mpk bid x 5d** | 1401 | 1461 | 201 | 241 | 48 | 6 | 6 | 1 | 1 |
|  | 701 | 501 | 221 | 28 | 5 | 2 | 2 | 1 | 1 |
|  | 1261 | 1141 | 361 | 135 | 45 | 3 | 8 | 1 | 1 |
|  | 721 | 621 | 241 | 54 | 28 | 2 | 1 | 1 | 1 |
|  | 621 | 481 | 39 | 78 | 19 | 4 | 3 | 1 | 1 |
|  | 841 | 2341 | 1341 | 621 | 381 | 58 | 30 | 3 | 1 |
| **Average** | 924.33 | 1091.00 | 400.67 | 192.83 | 87.67 | 12.50 | 8.33 | 1.33 | 1.00 |
| **SD** | 325.80 | 727.05 | 472.07 | 222.99 | 144.60 | 22.34 | 10.93 | 0.82 | 0.00 |
| **p (ANOVA)** | >0.9999 | 0.14 | <0.0001 | <0.0001 | 0.01 | 0.03 | 0.00 | 0.12 | 0.97 |
| **AWZ1066 50 mpk bid x5d** | 201 | 1421 | 1421 | 741 | 170 | 49 | 16 | 2 | 1 |
|  | 361 | 1821 | 3441 | 2541 | 841 | 201 | 70 | 5 | 3 |
|  | 981 | 89 | 1 | 2 | 1 | 1 | 1 | 1 | 1 |
|  | 321 | 2621 | 4761 | 1701 | 321 | 36 | 2 | 5 | 1 |
|  | 661 | 1181 | 4121 | 741 | 361 | 37 | 24 | 4 | 1 |
|  | 321 | 2221 | 3981 | 1801 | 341 | 11 | 1 | 1 | 1 |
| **Average** | 474.33 | 1559.00 | 2954.33 | 1254.50 | 339.17 | 55.83 | 19.00 | 3.00 | 1.33 |
| **SD** | 291.94 | 889.67 | 1843.33 | 921.87 | 281.29 | 73.33 | 26.73 | 1.90 | 0.82 |
| **p (ANOVA)** | 1.00 | 0.36 | 0.00 | <0.0001 | 0.03 | 0.03 | 0.00 | 0.12 | 0.97 |
| **AWZ1066 50 mpk bid + ABZ 10 mpk bid x 5d** | 241 | 241 | 50 | 7 | 1 | 1 | 1 | 1 | 1 |
|  | 1221 | 1221 | 281 | 19 | 13 | 1 | 1 | 1 | 1 |
|  | 201 | 16 | 5 | 37 | 7 | 2 | 1 | 1 | 1 |
|  | 441 | 341 | 97 | 36 | 6 | 4 | 1 | 1 | 1 |
|  | 601 | 661 | 1921 | 299 | 26 | 4 | 2 | 1 | 1 |
|  | 741 | 1421 | 1241 | 179 | 59 | 5 | 1 | 1 | 1 |
| **Average** | 574.33 | 650.17 | 599.17 | 96.17 | 18.67 | 2.83 | 1.17 | 1.00 | 1.00 |
| **SD** | 378.14 | 563.08 | 795.89 | 117.50 | 21.55 | 1.72 | 0.41 | 0.00 | 0.00 |
| **p (ANOVA)** | 1.00 | 0.05 | <0.0001 | <0.0001 | 0.01 | 0.03 | 0.00 | 0.12 | 0.97 |
| **AWZ1066 50 mpk bid x7d** | 201 | 301 | 921 | 145 | 2 | 1 | 1 | 1 | 1 |
|  | 201 | 561 | 861 | 521 | 67 | 13 | 6 | 2 | 1 |
|  | 135 | 761 | 641 | 401 | 14 | 1 | 1 | 1 | 1 |
|  | 461 | 641 | 861 | 158 | 39 | 6 | 2 | 2 | 1 |
|  | 221 | 1121 | 2881 | 1281 | 301 | 67 | 8 | 1 | 1 |
|  | 1261 | 1741 | 4841 | 2381 | 201 | 25 | 2 | 1 | 1 |
| **Average** | 413.33 | 854.33 | 1834.33 | 814.50 | 104.00 | 18.83 | 3.33 | 1.33 | 1.00 |
| **SD** | 430.22 | 510.63 | 1690.48 | 872.68 | 120.26 | 25.27 | 2.94 | 0.52 | 0.00 |
| **p (ANOVA)** | 1.00 | 0.08 | <0.0001 | <0.0001 | 0.02 | 0.03 | 0.00 | 0.12 | 0.97 |
| **ABZ 10 mpk bid x 5d** | 221 | 241 | 55 | 281 | 64 | 33 | 20 | 8 | 1 |
|  | 321 | 441 | 301 | 361 | 301 | 361 | 389 | 952 | 1041 |
|  | 221 | 601 | 661 | 261 | 80 | 5 | 5 | 28 | 9 |
|  | 241 | 361 | 281 | 221 | 119 | 42 | 44 | 24 | 281 |
|  | 1221 | 1061 | 581 | 521 | 261 | 3601 | 215 | 122 | 201 |
|  | 881 | 581 | 281 | 481 | 201 | 572 | 487 | 283 | 681 |
| **Average** | 517.67 | 547.67 | 360.00 | 354.33 | 171.00 | 769.00 | 193.33 | 236.17 | 369.00 |
| **SD** | 428.47 | 285.56 | 222.90 | 123.07 | 98.32 | 1405.66 | 206.31 | 365.53 | 412.29 |
| **p (ANOVA)** | 1.00 | 0.03 | <0.0001 | <0.0001 | 0.02 | 0.21 | 0.00 | 0.21 | 1.00 |
|  |  |  |  |  |  |  |  |  |  |

**Table S5: *L. sigmodontis* embryo counts post-treatment with AWZ1066S, ABZ or AWZ1066S+ABZ in gerbils**

Different embryonic stages from adult *L. sigmodontis* females were quantified and median and mean numbers with standard deviation (SD) and % reduction compared to vehicle group is enlisted in the table. Multiple embryonic stages were found to be significantly different between vehicle and drug treatment groups when analysed by Kruskal-Wallis 1-way-ANOVA (*p* value: eggs <0.0001; morula <0.0001; pretzel <0.0001; Stretched mf = 0.0004; degrading early embryos <0.0001; degrading late embryos <0.0001). Post-hoc Dunn’s test was carried out to identify specific differences between control and treated groups and p values obtained after the test is specified in below table [ANOVA (p)].

| **Treatment** | **Eggs+1**  **/worm** | **Morula+1**  **/worm** | **Pretzel+1**  **/worm** | **Streched mf +1 /worm** | **Degenerated early embryos +1 /worm** | **Degenerated late embryos+1 /worm** |
| --- | --- | --- | --- | --- | --- | --- |
| **Vehicle *bid* x 5d** | 18001 | 35001 | 18001 | 18001 | 1 | 1 |
|  | 16001 | 20001 | 18001 | 23001 | 1 | 1 |
|  | 28001 | 14001 | 26001 | 25001 | 1 | 1 |
|  | 36001 | 40001 | 18001 | 15001 | 1 | 1 |
|  | 27001 | 74001 | 28001 | 22001 | 1 | 1 |
|  | 45001 | 19001 | 36001 | 1 | 1 | 1 |
|  | 20001 | 24001 | 11001 | 1001 | 1 | 1 |
|  | 35001 | 19001 | 16001 | 1 | 1 | 1 |
|  | 20001 | 75001 | 1 | 1 | 1 | 1 |
|  | 48001 | 71001 | 21001 | 23001 | 1 | 1 |
|  | 53001 | 75001 | 49001 | 27001 | 1 | 1 |
|  | 59001 | 122001 | 55001 | 21001 | 1 | 1 |
|  | 4001 | 16801 | 1 | 401 | 1 | 1 |
| Median | 28001 | 35001 | 18001 | 18001 | 1 | 1 |
| Mean | 31463 | 46524 | 22770 | 13493 | 1 | 1 |
| SD | 16282 | 33560 | 16412 | 11257 | 0 | 0 |
| **ABZ 10mg/kg *bid* x5d** | 1001 | 45001 | 5001 | 1001 | 1 | 1 |
|  | 3201 | 6001 | 101 | 1 | 201 | 1 |
|  | 12001 | 37001 | 9001 | 10001 | 1001 | 1 |
|  | 6001 | 48001 | 17001 | 7001 | 1001 | 1 |
|  | 11001 | 110001 | 6001 | 9001 | 1 | 1 |
|  | 25001 | 100001 | 19001 | 28001 | 1 | 1 |
|  | 30001 | 150001 | 24001 | 33001 | 1 | 1 |
|  | 44001 | 149001 | 25001 | 57001 | 1 | 1 |
|  | 8101 | 27601 | 1601 | 2001 | 1 | 1 |
|  | 1541 | 231 | 1 | 11 | 11 | 1 |
|  | 11001 | 44001 | 17001 | 46001 | 1001 | 3001 |
|  | 15001 | 84001 | 7001 | 7001 | 1001 | 16001 |
|  | 35001 | 7001 | 1 | 1 | 1 | 1 |
| Median | 11001 | 45001 | 7001 | 7001 | 1 | 1 |
| Mean | 15604 | 62142 | 10055 | 15386 | 325 | 1463 |
| SD | 13685 | 51752 | 9217 | 19246 | 472 | 4446 |
| % reduction | 60.7 | -37.1 | 6.1 | 61.1 | 0 | 0 |
| ANOVA (p) | ns | ns | ns | ns | ns | ns |
| **AWZ1066S 25mg/kg *bid* x5d** | 21001 | 68001 | 42001 | 16001 | 10001 | 6001 |
|  | 45001 | 139001 | 31001 | 46001 | 1001 | 8001 |
|  | 5001 | 23001 | 18001 | 51001 | 2001 | 1 |
|  | 6401 | 7001 | 1 | 601 | 1 | 1 |
|  | 7301 | 17201 | 1 | 1 | 1 | 1 |
|  | 18001 | 62001 | 41001 | 20001 | 1 | 8001 |
|  | 81 | 61 | 2711 | 1 | 161 | 61 |
|  | 1101 | 201 | 101 | 1 | 3801 | 301 |
|  | 13101 | 29801 | 2201 | 1 | 301 | 1901 |
|  | 31 | 14 | 7 | 3 | 45 | 18 |
|  | 9001 | 37001 | 14001 | 1001 | 6001 | 12001 |
|  | 21001 | 75001 | 1001 | 1 | 3001 | 2001 |
|  | 1 | 301 | 1 | 1 | 21301 | 601 |
|  | 101 | 401 | 1 | 1 | 11701 | 701 |
|  | 31 | 31 | 11 | 11 | 621 | 81 |
|  | 1 | 101 | 1 | 1 | 14401 | 201 |
|  | 5001 | 33001 | 1701 | 1 | 7001 | 1 |
|  | 16001 | 58001 | 13001 | 1001 | 1 | 5001 |
|  | 10101 | 21201 | 5001 | 1 | 3701 | 901 |
| Median | 6401 | 21201 | 1701 | 1 | 2001 | 601 |
| Mean | 9382 | 30070 | 9039 | 7138 | 4476 | 2409 |
| SD | 11341 | 36719 | 14111 | 15648 | 5987 | 3592 |
| % reduction | 77.1 | 39.4 | 87.7 | 99.99 | -200000 | -60000 |
| ANOVA (p) | 0.02 | ns | ns | ns | 0.0003 | 0.0001 |
| **AWZ1066S 25mg/kg *bid* x5d + ABZ 10mg/kg *bid* x5d** | 11 | 11 | 21 | 1 | 1041 | 81 |
|  | 78 | 13 | 4 | 3 | 5 | 12 |
|  | 81 | 1 | 1 | 1 | 371 | 91 |
|  | 101 | 61 | 1 | 1 | 711 | 101 |
|  | 291 | 611 | 1 | 1 | 61 | 11 |
|  | 5801 | 10501 | 2901 | 101 | 3701 | 1301 |
|  | 51 | 41 | 1 | 1 | 461 | 71 |
|  | 301 | 1 | 101 | 101 | 13401 | 501 |
|  | 101 | 1 | 101 | 1 | 5801 | 101 |
|  | 4301 | 5401 | 1 | 1 | 1 | 1 |
|  | 91 | 11 | 201 | 11 | 231 | 411 |
| Median | 101 | 13 | 4 | 1 | 461 | 91 |
| Mean | 1019 | 1514 | 303 | 20 | 2344 | 244 |
| SD | 2024 | 3385 | 864 | 40 | 4105 | 387 |
| % reduction | 99.6 | 99.96 | 99.98 | 99.99 | -46000 | -9000 |
| ANOVA (p) | <0.0001 | 0.0005 | 0.01 | ns | 0.008 | 0.007 |
| **AWZ1066S 50mg/kg bid x7d** | 301 | 301 | 1 | 1 | 6801 | 1 |
|  | 101 | 201 | 1 | 1 | 36601 | 301 |
|  | 101 | 101 | 1 | 1 | 8201 | 1 |
|  | 1 | 201 | 1 | 1 | 20401 | 1 |
| Median | 101 | 201 | 1 | 1 | 14301 | 1 |
| Mean | 126 | 201 | 1 | 1 | 18001 | 76 |
| SD | 126 | 82 | 0 | 0 | 13823 | 150 |
| % reduction | 99.6 | 99.4 | 99.99 | 99.99 | -1430000 | 0 |
| ANOVA (p) | 0.004 | ns | 0.01 | ns | <0.0001 | ns |
| **AWZ1066S 50mg/kg bid x5d** | 101 | 1 | 1 | 101 | 7001 | 1 |
| **AWZ1066S 50mg/kg bid x5d + ABZ 10mg/kg bid x5d** | No worms to analyse | | | | | |

**References:**

1. Turner JD, Sharma R, Al Jayoussi G, Tyrer HE, Gamble J, Hayward L, et al. Albendazole and antibiotics synergize to deliver short-course anti-Wolbachia curative treatments in preclinical models of filariasis. Proc Natl Acad Sci U S A. 2017;114(45):E9712-E21. Epub 2017/10/29. doi: 10.1073/pnas.1710845114. PubMed PMID: 29078351; PubMed Central PMCID: PMCPMC5692564.
